# Enhanced Philoponella Prominens Optimization (EESPPO) Algorithm Integrated with Experience Exchange Strategy for Global Optimization and Engineering Design Problems

**DOI:** 10.3390/biomimetics11060407

**Published:** 2026-06-09

**Authors:** Zhongzhen Yan, Yi Yu, Yuan Cao, Jie Gao

**Affiliations:** School of Computer Science and Artificial Intelligence, Hubei University of Technology, Wuhan 430068, China; 102411213@hbut.edu.cn (Y.C.); 102411234@hbut.edu.cn (J.G.)

**Keywords:** Philoponella Prominens Optimization (PPO), bio-inspired meta-heuristics, experience exchange strategy, global optimization, population diversity, engineering design optimization, CEC2017 benchmark

## Abstract

To address the challenges of high-dimensional nonlinearity, multimodal landscapes, and stringent constraints prevalent in modern engineering design, traditional meta-heuristic algorithms often suffer from a loss of population diversity and premature convergence. Inspired by the social collaborative predation and collective information interaction behaviors of *P. prominens* (jumping spiders), this study proposes a novel bio-inspired meta-heuristic optimization algorithm, termed the Experience Exchange Strategy-Enhanced Philoponella Prominens Optimization (EESPPO). The proposed EESPPO integrates an Experience Exchange Strategy framework to reshape the search dynamics of the population through three progressive evolutionary stages: (1) In the Experience Scarcity (ESC) stage, the algorithm focuses on the construction and dynamic maintenance of an experience library to ensure the effective preservation of high-quality historical information; (2) In the Experience Crossover (ECR) stage, a random guidance vector generation mechanism is introduced to significantly enhance population behavioral diversity and the capability to escape local optima; (3) In the Experience Sharing (ESH) stage, an adaptive fusion update strategy is employed to achieve efficient information interaction and co-evolution among individuals. These three stages operate synergistically within the optimization cycle to establish a dynamic balance between global exploration and local exploitation, effectively overcoming the inherent defects of premature convergence in traditional meta-heuristics. Extensive empirical analysis based on the CEC2017 benchmark functions confirms that EESPPO comprehensively outperforms 12 existing advanced algorithms (including PPO, HSO, SGA, PSO, FLO, DE, HO, WOA, KEO, GWO, FDB-AGSK, and IVYPSO) in terms of convergence accuracy and robustness. Furthermore, the application of EESPPO to four challenging engineering design problems confirms its superiority. The experimental results validate the high precision and feasibility of EESPPO in solving complex constrained engineering problems.

## 1. Introduction

In modern scientific research and industrial production, finding optimal parameters to maximize system efficiency or minimize operational costs is a fundamental yet formidable challenge. As technological systems become increasingly sophisticated, the optimization problems associated with them grow exponentially in complexity [1]. These real-world tasks typically involve massive search spaces, non-convexity, and highly restrictive physical constraints, rendering intuitive trial-and-error or conventional analytical approaches practically obsolete. Following this general trend, such intricate optimization problems are ubiquitously distributed across specific advanced domains, spanning machine learning [2,3,4], path planning [5], engineering design [6,7], resource allocation [8], image segmentation [9], and complex control systems [10]. However, when tackling these complex problems—which are typically characterized by high dimensionality, nonlinearity, non-convexity, and multimodal landscapes—traditional mathematical optimization methods [11,12,13], such as gradient-based techniques [14] and linear programming [15], often prove inadequate. These conventional approaches are not only computationally expensive but also highly sensitive to initial conditions, making them susceptible to stagnation in local optima. Furthermore, they struggle to solve for global optima in the presence of dynamic or discontinuous search spaces. To overcome these severe challenges, various meta-heuristic algorithms have emerged [16,17]. By virtue of their gradient-free nature, flexible mechanisms, and robust global search capabilities, meta-heuristics have become indispensable tools for addressing complex optimization tasks [18,19].

Over the past decades, Swarm Intelligence (SI), a prominent branch of meta-heuristics, has witnessed the emergence of numerous classic paradigms [20,21,22,23]. Particle Swarm Optimization (PSO) simulates the social collaboration and information sharing of bird flocks to guide individuals toward optimal solutions [24]. Differential Evolution (DE) drives the evolution of candidate solutions based on population mutation, crossover, and selection operations [25]. Grey Wolf Optimizer (GWO) mimics the strict social hierarchy and pack hunting strategies of grey wolves [26], while the Whale Optimization Algorithm (WOA) was inspired by the unique bubble-net feeding behavior of humpback whales [27]. As research progressed, advanced hybrid and enhancement strategies were proposed to further improve performance in complex landscapes [28,29,30]. For instance, the FDB-AGSK algorithm cleverly integrates a Fitness-Distance Balance mechanism with an adaptive knowledge acquisition and sharing strategy, significantly bolstering its global exploration capabilities [31].

Recently, the field has experienced a renewed surge of innovation, yielding several high-performance algorithms with unique mechanisms [32,33,34]. Among the algorithms proposed in 2024, the Frilled Lizard Optimization (FLO) vividly simulates the “sit-and-wait” predation strategy and the iconic bipedal escape behavior of frilled lizards [35]. The Hippo Optimization (HO) algorithm establishes a mathematical model based on the defensive position updating and aggressive behaviors of hippos in rivers [36]. Moving into 2025, the sources of inspiration for algorithm design have become even more diverse: Holistic Swarm Optimization (HSO) focuses on simulating the holistic emergent behavior of biological groups to achieve efficient collaborative optimization [37]; Schrödinger Guest Algorithm (SGA) innovatively introduces the Schrödinger equation from physics and crystal growth dynamics into the optimization process [38]; Kangaroo Escape Optimization (KEO) emphasizes the survival escape and jumping mechanisms of kangaroos when facing predators [39]; meanwhile, IVYPSO combines the growth characteristics of ivy plants with the guidance mechanism of PSO, demonstrating the powerful potential of hybrid algorithms [40].

Despite the proliferation of existing algorithms, the “No Free Lunch” (NFL) theorem [41] asserts that no single algorithm can perform optimally across all possible optimization problems. Philoponella Prominens Optimization (PPO) is an emerging algorithm that simulates the social collaborative predation behavior of jumping spiders [42]. Although it has shown promising potential in preliminary tests, it still suffers from inherent defects, such as insufficient information interaction among individuals and a tendency to fall into local optima when facing high-dimensional multimodal problems [43,44]. To break through these bottlenecks and fully exploit the potential of PPO, this paper proposes an enhanced optimization framework: EESPPO. The core motivation of this study lies in introducing an Experience Exchange Strategy (EES) based on the evolutionary state of the population [45], reshaping the search dynamics to balance global exploration and local exploitation. To clearly delineate the innovations and value of this research, the main contributions are summarized as follows:

- **Development of an enhanced EESPPO algorithm:** This algorithm innovatively integrates the Experience Exchange Strategy (EES), establishing a dynamic balance between exploration and exploitation through the dynamic experience library construction in the Experience Scarcity (ESC) stage, the random-guided escape mechanism in the Experience Crossover (ECR) stage, and the adaptive fusion update in the Experience Sharing (ESH) stage.
- **Comprehensive benchmarking and statistical validation:** The performance of EESPPO was extensively evaluated using the CEC2017 test suite. Through comparison with 12 state-of-the-art meta-heuristics, combined with Friedman rank ordering and Wilcoxon signed-rank tests, the significant advantages of EESPPO in terms of convergence accuracy, speed, and robustness were rigorously verified from a statistical perspective.
- **Resolution of challenging engineering constrained problems:** EESPPO was applied to four real-world engineering problems: gas transmission compressor system design, welded beam design, speed reducer design, and tension/compression spring design. In all experiments, the algorithm accurately converged to the theoretical global optima and demonstrated remarkable stability in handling complex physical constraints, proving its utility as a highly reliable engineering design tool.

The remainder of this paper is organized as follows: Section 2 briefly reviews the fundamental principles and mathematical model of the PPO algorithm. Section 3 details the proposed EESPPO enhancement framework, focusing on the three progressive stages of the Experience Exchange Strategy (EES) and their specific implementation mechanisms. Section 4 reports the comprehensive experimental results and analysis. Section 5 summarizes the key findings and outlines future research directions.

## 2. Philoponella Prominens Optimization (PPO)

### 2.1. Biological Inspiration and Conceptual Model

*Philoponella prominens* (family Uloboridae) is a unique social arthropod widely distributed in East Asia, typically inhabiting large communal web systems. These spiders exhibit highly specialized biological behaviors, most notably a remarkable survival strategy evolved by males to counter the risk of sexual cannibalism: post-mating catapulting. In nature, the moment mating concludes is the most perilous for the male. To evade female predation, the male *P. prominens* utilizes a hydraulic mechanism at the tibia-metatarsus joint to store immense elastic potential energy, instantaneously catapulting itself away at extreme acceleration. This behavior is characterized by high velocity and high-speed mid-air rotation, allowing the male to swiftly and stochastically escape danger zones, thereby significantly enhancing survival probability.

These biological traits inspired PPO, which simulates courtship, escape, and dual-track evolution (predation or rebirth) in a numerical optimization context. The algorithm integrates an exploration strategy mimicking explosive catapulting, a survival threshold-based game mechanism, and two distinct evolutionary paths: sexual cannibalism/rebirth and predation/exploitation. This combined mechanism enables PPO to dynamically adjust its strategy, effectively balancing global exploration and local exploitation, making it particularly suitable for complex, high-dimensional, and multimodal optimization problems.

### 2.2. Mathematical Model

PPO simulates the dynamic interactions of a population within the search space, primarily encompassing initialization, energy calculation, ejection escape, survival discrimination, and memory updates.

#### 2.2.1. Population Initialization and Spatial Modeling

PPO begins by generating an initial population of N male individuals. Each individual consists of D decision variables and follows a uniform distribution within a predefined search space:

(1) $$x_{i,j}^{0}=L_{j}+rand\cdot \left(U_{j}-L_{j}\right),\;i=1,\ldots ,N,\;j=1,\ldots ,D$$

(2) $$M_{i,j}^{0}=x_{i,j}^{0}$$

where $x_{i,j}^{0}$ denotes the spatial position of the i-th male in the j-th dimension at the initial moment; $U_{j}$ and $L_{j}$ represent the upper and lower bounds of the j-th decision variable, respectively; and rand is a random number between [0, 1]. Simultaneously, the algorithm initializes a historical memory population M to guide the subsequent evolution.

#### 2.2.2. Energy Calculation and Pairing

PPO utilizes bio-energy assessment and random pairing strategies to simulate the survival of the fittest and social interaction during the spider’s reproductive process. Males quantify their survival energy based on their current fitness relative to the population and establish dynamic links with the historical memory library:

(3) $$E_{i}^{t}=\frac{F_{max}^{t}+F_{min}^{t}-f\left(X_{i}^{t}\right)}{F_{max}^{t}+\epsilon }$$

(4) $$Y^{t}=Permutation\left(M^{t}\right)$$

where $F_{max}^{t}$ and $F_{min}^{t}$ represent the fitness boundaries in the current iteration. ϵ is an infinitesimally small constant (typically 10^16^, i.e., machine epsilon) used exclusively to prevent division-by-zero errors during computation. It serves strictly as a fixed numerical stabilizer rather than a tunable hyperparameter; thus, its value remains constant regardless of the problem’s specific characteristics or scale. $M^{t}$ is the historical memory population. Equation (4) indicates that the female’s position is a random permutation of the males’ historical best positions. By combining potential energy quantification with historical gene recombination, a foundation is laid for the algorithm to escape from local optima traps.

#### 2.2.3. Catapulting Escape Update

**Figure 1:**
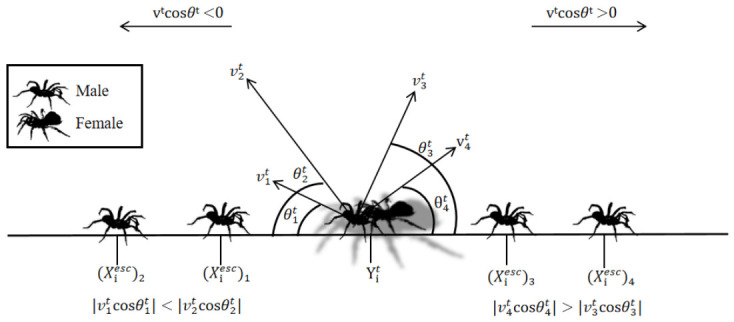
Catapulting the escape behavior of males.

PPO employs a catapulting escape strategy to simulate the explosive jumping behavior of male spiders after mating. Utilizing the bio-energy obtained in the previous stage and the relative distance from the mate, males generate escape momentum and execute a non-linear transition in the solution space:

(5) $$v_{i}^{t}=E_{i}^{t}\cdot ||X_{i}^{t}-Y_{i}^{t}||$$

(6) $$X_{i}^{esc}=Y_{i}^{t}+cos\left(\pi \cdot r_{1}\right)\cdot v_{i}^{t}$$

where $v_{i}^{t}$ represents the instantaneous catapulting velocity of the i-th individual, and the cos function is used to simulate the rotation trajectory of the spider in the air. As shown in Figure 1, this energy-driven stochastic oscillation mechanism maintains efficient global exploration, effectively avoiding premature convergence in the mid-iteration phase.

#### 2.2.4. Survival Discrimination and Dual-Track Evolution

PPO introduces a dynamic survival threshold determination mechanism. The algorithm first calculates the new Euclidean distance $D_{i}^{t}$ between the catapulted individual and its mate, and sets an adaptive threshold $\sigma^{t}$ that decays linearly:

(7) $$D_{i}^{t}=||X_{i}^{esc}-Y_{i}^{t}{\left|\right|}_{2}$$

(8) $$\sigma^{t}=\mu \left(D^{t}\right)\cdot \left[\left(1-\frac{t}{T_{max}}\right)+0.5\right]$$

where $\mu \left(D^{t}\right)$ represents the mean value of paired distances, and $T_{max}$ is the maximum number of iterations. Based on the comparison between $D_{i}^{t}$ and $\sigma^{t}$, two distinct position update strategies are activated:

(9) $$X_{i}^{t+1}=\left\{\begin{array}{lll} Y_{i}^{new}+Levy\left(\beta \right)\cdot \omega^{t}\cdot exp\left(1-\frac{t}{T_{max}}\right), & if\;D_{i}^{t}<\sigma^{t} & \\ X_{best}^{t}+cos\left(\pi \cdot r_{3}\right)\cdot \left(X_{i}^{esc}-X_{best}^{t}\right), & if\;D_{i}^{t}\geq \sigma^{t} & \end{array}\right.$$

(1) **Sexual Cannibalism and Rebirth (**$D_{i}^{t}$ **<** $\sigma^{t}$**):** The male fails to escape. The mate undergoes a fine-tuning adjustment $Y_{i}^{new}$, and the individual’s position is replaced via Lévy flight:

(10) $$Y_{i}^{new}=Y_{i}^{t}+r_{2}\cdot E_{i}^{t}\cdot \left(X_{i}^{esc}-Y_{i}^{t}\right)$$

(11) $$Levy\left(\beta \right)=\frac{u\cdot \phi }{{\left|v\right|}^{1/\beta }},\;where\;\phi ={\left[\frac{\Gamma \left(1+\beta \right)\cdot sin\left(\frac{\pi \beta }{2}\right)}{\Gamma \left(\frac{1+\beta }{2}\right)\cdot \beta \cdot 2^{\frac{\beta -1}{2}}}\right]}^{\frac{1}{\beta }}$$

This mechanism prevents stagnation in local optima through mandatory random mutation.

(2) **Predation and Exploitation (**$D_{i}^{t}$ **>** $\sigma^{t}$**):** The male successfully survives and initiates a spiral approach toward the global optimum $X_{best}^{t}$, ensuring fine-tuned exploitation capability.

#### 2.2.5. Memory Population Update

A greedy selection strategy is adopted to ensure the monotonic optimization of historical information:

(12) $$M_{i}^{t+1}=\left\{\begin{array}{lll} X_{i}^{t+1}, & if\;f\left(X_{i}^{t+1}\right)<f\left(M_{i}^{t}\right) & \\ M_{i}^{t}, & otherwise & \end{array}\right.$$

where $Up_{c}$ and $Low_{c}$ define the local bounds of the optimal “eggs,” and fitness comparison determines whether individuals update their positions.

### 2.3. Section Summary

**Figure 2:**
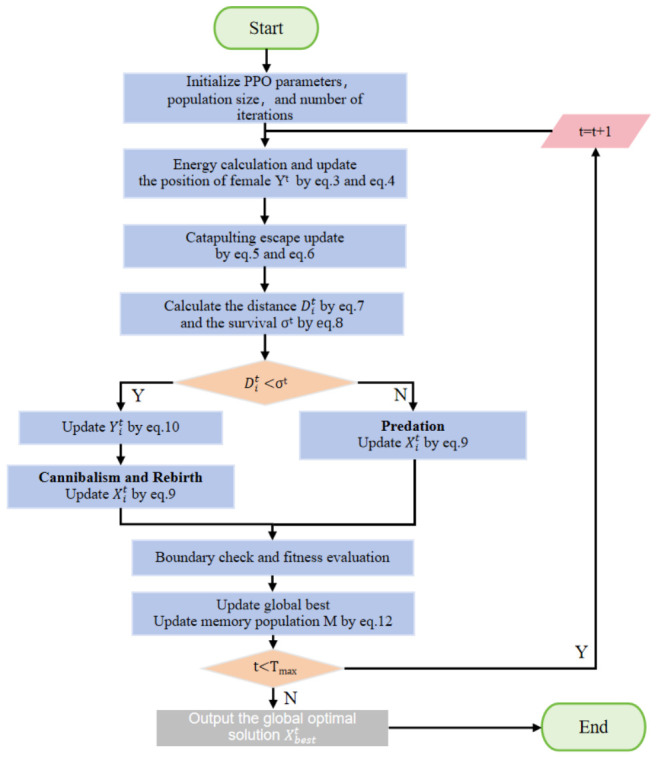
Flowchart of PPO.

The flowchart of PPO is presented in Figure 2. By integrating bio-energy quantification, non-linear ejecting, and dual-track evolution, PPO effectively circumvents local extrema while achieving high-precision convergence, establishing its position as a highly competitive meta-heuristic solver.

## 3. Improved PPO Algorithm Integrating Experience Exchange Strategy (EESPPO)

The Philoponella Prominens Optimization algorithm simulates the unique social behaviors of jumping spiders, including the catapulting mechanism for global exploration and the dual-track evolutionary strategy for local exploitation. However, when dealing with high-dimensional and complex optimization landscapes, the population is prone to falling into local optima due to the lack of effective guidance from historical information. To overcome this drawback, this chapter proposes an enhanced algorithm: the Improved Philoponella Prominens Optimization integrated with Experience Exchange Strategy (EESPPO). This algorithm introduces a probability-triggered Experience Exchange Strategy (EES). By formalizing EES into three mathematical processes—dynamic maintenance of an experience pool, stochastic guidance generation, and adaptive information fusion—and seamlessly embedding them into the iterative loop of PPO, effective guidance of current searches by high-quality historical experience is achieved.

### 3.1. Integration of Experience Exchange Strategy

In EESPPO, the Experience Exchange Strategy is constructed as a closed-loop system comprising three progressive stages: the Experience Scarcity (ESC) stage, the Experience Crossover (ECR) stage, and the Experience Sharing (ESH) stage.

#### 3.1.1. Construction and Dynamic Maintenance of the Experience Pool (ESC Stage)

In meta-heuristic algorithms, high-quality solutions discovered during the early search phase are often scarce and can be easily lost as the population continues to evolve. To address this, the algorithm establishes an external archive—referred to as the Experience Pool (denoted by E)—to protect and store these valuable historical positions, thereby providing reliable guidance for subsequent iterations.

The first step is to determine the appropriate size of this pool. Let N represent the total population size and K represent a predefined maximum capacity threshold for the experience pool. To prevent the pool’s size from exceeding the actual population size (which could cause redundancy), its operational size, denoted as $N_{ep}$, is strictly defined as follows:

(13) $$N_{ep}=min\left(K,N\right)$$

During the t-th iteration, the experience pool set can be formally represented as a collection containing $N_{ep}$ experience vectors:

(14) $$E^{t}=\{e_{1}^{t},e_{2}^{t},\ldots ,e_{N_{ep}}^{t}\}$$

where $e_{k}^{t}\in R^{D}$ denotes the position vector of the K-thhistorical elite individual in the D-dimensional search space. 

Initialization Process: At the very beginning of the optimization process (t=0), the algorithm evaluates the initial random population $X^{0}$. To select the initial experiences, the individuals are sorted in ascending order based on their fitness values $F\left(\cdot \right)$ (assuming a minimization problem, where a lower fitness value represents a better solution). The top $N_{ep}$ superior individuals with the lowest fitness values are then extracted to construct the initial experience pool:

(15) $$E^{0}=\{x_{\left(i\right)}^{0}|F\left(x_{\left(1\right)}^{0}\right)\leq F\left(x_{\left(2\right)}^{0}\right)\leq \ldots \leq F\left(x_{\left(N\right)}^{0}\right),1\leq i\leq N_{ep}\}$$

Dynamic Maintenance Mechanism: As the search progresses, the population will inevitably discover new solutions that are superior to those currently stored. To ensure the experience pool always retains the most competitive historical information, a greedy elimination strategy is employed. First, the algorithm identifies the “worst” experience currently in the pool (i.e., the individual with the highest fitness value). The index of this worst individual, defined as $k_{worst}$, is identified by:

(16) $$k_{worst}=arg\underset{k\in \{1,\ldots ,N_{ep}\}}{max}F\left(e_{k}^{t}\right)$$

Next, the algorithm compares the fitness of any newly generated individual $x_{i}^{t}$ in the current population against the worst experience. If the new individual exhibits a better (lower) fitness value, it directly replaces $e_{k_{worst}}^{t}$ in the pool. The update rule for the experience pool is thus defined as follows:

(17) $$E^{t+1}=\left\{\begin{array}{lll} \left(E^{t}\backslash \{e_{k_{worst}}^{t}\}\right)\cup \{x_{i}^{t}\}, & if\;F\left(x_{i}^{t}\right)<F\left(e_{k_{worst}}^{t}\right) & \\ E^{t}, & otherwise & \end{array}\right.$$

Equation (17) functions as a strict survival-of-the-fittest filter. It guarantees that the experience pool E continuously discards outdated, inferior information while stably assimilating the scarce, high-quality solutions generated throughout the evolutionary process.

#### 3.1.2. Generation of Random Guidance Vectors (ECR Stage)

In many optimization scenarios, relying solely on the current population’s trajectory can lead to “single-path dependency,” where individuals prematurely converge toward a local optimum. To prevent this and inject vital behavioral diversity into the swarm, the Experience Crossover (ECR) stage introduces a stochastic perturbation mechanism.

Instead of applying this perturbation to every individual unconditionally—which might severely disrupt the algorithm’s convergence—a probabilistic trigger is employed. A Bernoulli random variable $I_{ees}$ is introduced to dictate the activation of the strategy, parameterized by a predefined trigger probability, $P_{ees}$:

(18) $$I_{ees}∼Bernoulli\left(P_{ees}\right)$$

Here, the variable $I_{ees}$ essentially acts as a logical switch (a coin flip). When $I_{ees}=1$, the ECR stage is initiated.

To generate a new, promising search direction, the algorithm looks back into the Experience Pool E (constructed in the ESC stage). It randomly selects two distinct indices, $r_{1}$ and $r_{2}$, following a discrete uniform distribution bounded by the pool’s size $N_{ep}$:

(19) $$r_{1},r_{2}∼U\{1,N_{ep}\},\;s.t.r_{1}\neq r_{2}$$

It is crucial that the selected indices are strictly distinct ($r_{1}\neq r_{2}$). This constraint ensures that the subsequent recombination occurs between two completely different spatial positions, thereby maximizing the diversity of the generated information and preventing stagnant, self-repeating updates.

Based on the two selected historical elite experiences, $e_{r_{1}}^{t}$ and $e_{r_{2}}^{t}$, a new experience guidance vector, $G_{i}^{t}$, is constructed for the current individual i. This vector is generated via a linear weighted crossover operation:

(20) $$G_{i}^{t}=\lambda \cdot e_{r_{1}}^{t}+\left(1-\lambda \right)\cdot e_{r_{2}}^{t}$$

In this equation, λ is a random weighting factor drawn from a standard continuous uniform distribution $\;U\left(0,1\right)$. Geometrically, Equation (20) performs a random interpolation along the line segment connecting the two historical high-quality solutions. Because both endpoints of this segment represent superior locations previously discovered by the swarm, the intermediate subspace spanned between them possesses a highly probable exploration value. By exploring this “fertile” region, the ECR stage efficiently generates novel directions that robustly assist the population in escaping local optima.

#### 3.1.3. Adaptive Fusion Update (ESH Stage)

Once the new guidance vector $G_{i}^{t}$ is generated, the individual must decide how much to trust this new direction versus its original position. This decision is handled in the Experience Sharing (ESH) stage. At this juncture, the final position of the individual, denoted as $x_{i}^{new}$, is determined by a weighted convex combination of its current position $x_{i}^{t}$ and the guidance vector $G_{i}^{t}$:

(21) $$x_{i}^{new}=\alpha \left(t\right)\cdot G_{i}^{t}+\left(1-\alpha \left(t\right)\right)\cdot x_{i}^{t}$$

Intuitively, Equation (21) acts as a positional compromise between self-preservation and social learning. The parameter $\alpha \left(t\right)$ serves as a time-varying dynamic fusion factor. A higher $\alpha \left(t\right)$ pulls the individual closer to the shared experience $G_{i}^{t}$, whereas a lower $\alpha \left(t\right)$ keeps the individual nearer to its original location $x_{i}^{t}$.

In meta-heuristic optimization, a fundamental principle is to encourage broad global exploration in the early stages and fine-grained local exploitation in the later stages. To strictly align with this evolutionary rhythm, $\alpha \left(t\right)$ cannot be a static value; rather, it is designed as a linear decay function dependent on the iteration count:

(22) $$\alpha \left(t\right)=\alpha_{base}+\alpha_{var}\cdot \left(1-\frac{t}{T_{max}}\right)$$

In Equation (22), $T_{max}$ represents the maximum number of iterations. The parameter $\alpha_{base}$ denotes the base retention weight—this acts as the “floor” or minimum fusion ratio, ensuring that individuals never completely ignore the swarm’s shared experience even at the very end of the optimization. Meanwhile, $\alpha_{var}$ signifies the amplitude of weight variation, providing an extra boost of guidance during the initial exploration phase.

Through the synergistic effect of Equations (21) and (22), the algorithm’s search behavior is dynamically sculpted. At the beginning of the search (t→0), the fusion factor reaches its maximum ($\alpha \left(t\right)\to \alpha_{base}+\alpha_{var}$); this strong guidance forces individuals to take large strides toward the collective experience, thereby promoting global dispersion and preventing early stagnation. Conversely, as the search approaches termination ($t\to T_{max}$), the fusion factor decays to its minimum ($\alpha \left(t\right)\to \alpha_{base}$); this weak guidance allows individuals to focus primarily on refining their immediate surroundings, thereby assisting in precise local convergence.

### 3.2. Pseudocode and Process Description of EESPPO

**Figure 3:**
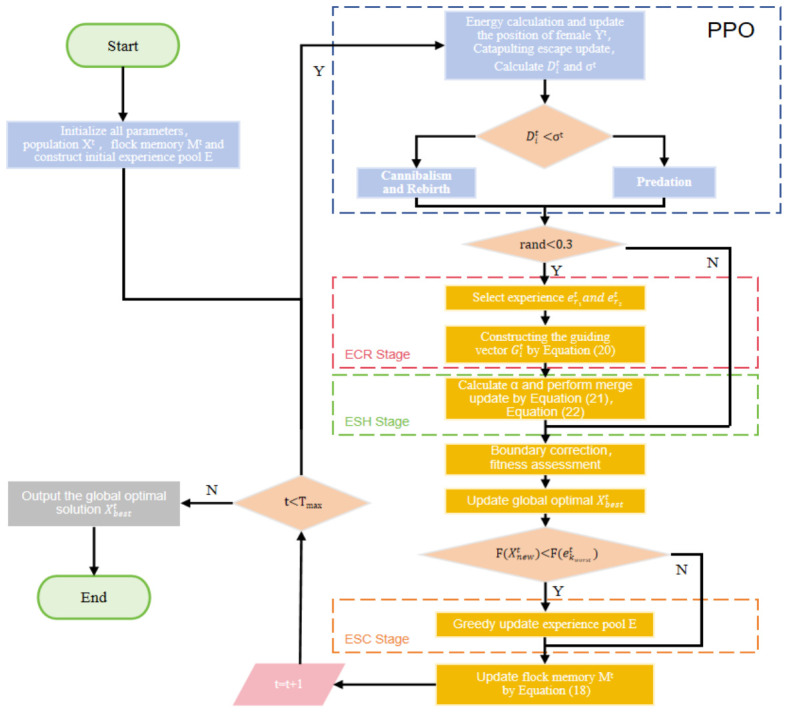
Flowchart of EESPPO.

To clearly elucidate the logical structure of EESPPO, Algorithm 1 provides a detailed pseudocode description, while the corresponding flowchart is illustrated in Figure 3. Addressing the deficiency of the standard PPO algorithm in easily becoming trapped in local optima within complex multi-modal landscapes, this framework introduces a multi-stage experience exchange mechanism to enhance search robustness.

The optimization process commences with the random initialization of the population within the search space, followed by the construction of the initial experience pool and population memory based on fitness evaluation results. Upon entering the main iteration loop, the algorithm first calculates the energy state and survival threshold of each individual. Subsequently, individuals execute the jumping operation and dynamically select an evolutionary path based on their distance to the mating target: if the distance is less than the threshold, a cannibalism strategy combined with Lévy flights is executed to enhance perturbation; otherwise, a spiral approach predation strategy is employed for fine-grained exploitation.

Immediately following this, the experience exchange strategy is conditionally triggered based on a preset probability. Once activated, the algorithm extracts superior historical information from the experience pool to construct a guidance vector and updates the individual’s position via a dynamic fusion factor, effectively breaking evolutionary stagnation. After completing the position update, the algorithm not only performs boundary constraint checks and updates the global optimum but, crucially, dynamically maintains the experience pool through the experience scarcity mechanism. This involves replacing inferior experiences in the pool with newly generated high-quality solutions and employing a greedy strategy to refresh the population memory. This process iterates continuously until the termination conditions are met, ultimately outputting the global optimal solution.
**Algorithm 1.** EESPPOInput: N: Population size T_max: Maximum iterations D: Problem dimension, [L, U]: Boundaries P_ees: Experience exchange probability (e.g., 0.3) N_ep: Capacity of Experience Pool (e.g., 20)   Output: x_best: Global best solution   1: // --- Initialization Phase --- 2: Initialize population X randomly within [L, U] 3: Evaluate fitness F(X), identify x_best 4: Sort X based on fitness to construct initial Experience Pool E (Equation (16)) 5: Initialize Flock Memory M = X 6: 7: // --- Main Evolution Loop --- 8: While (t < T_max) do 9:   Calculate Energy E_i for all individuals 10:   Generate Pairing Y by randomly permuting M 11:   Calculate Survival Threshold σ^t 12:   13:   For i = 1 to N do 14:     // [Part 1: Standard PPO Dynamics] 15:     Calculate Ejection Velocity v_i 16:     Perform Ejection to get intermediate position x_i (Equation (6) in Section 2) 17:     Calculate Distance D_i = ||x_i − Y_i|| 18:     19:     If D_i < σ^t then 20:       Update x_i via Cannibalism & Levy Flight (Equation (9) in Section 2) 21:     Else 22:       Update x_i via Predation (Spiral approach) (Equation (9) in Section 2) 23:     End If 24:     25:     // [Part 2: EES Strategic Enhancement] 26:     If rand() < P_ees then 27:       Select distinct indices r1, r2 from Experience Pool E 28:       Construct Guide Vector G_i^t (Equation (20)) 29:       Calculate dynamic fusion factor α(t) (Equation (22)) 30:       Update x_i = α(t) * G_i^t + (1 − α(t)) * x_i (Equation (21)) 31:     End If 32:     33:     // [Part 3: Evaluation & Maintenance] 34:     Check and correct boundary constraints for x_i 35:     Calculate fitness F_new = f(x_i) 36:     37:     // Update Global Best 38:     If F_new < f(x_best) then x_best = x_i 39:     40:     // Update Experience Pool (ESC Mechanism) 41:     Find worst experience e_worst in E 42:     If F_new < f(e_worst) then 43:       Replace e_worst with x_i (Equation (17)) 44:     End If 45:   End For 46:   47:   // Update Flock Memory 48:   Update M using Greedy Selection Strategy 49:   t = t + 1 50: End While 51: Return x_best


### 3.3. Time Complexity Analysis of EESPPO

Time complexity serves as a core metric for evaluating the operational efficiency of an algorithm. Let N denote the population size, D the problem dimension, $T_{max}$ the maximum number of iterations, and $C_{obj}$ the cost of a single objective function evaluation. The computational overhead of EESPPO is primarily constituted by two components: initialization and iterative updates.

The time complexity of the initialization phase is $O\left(N\cdot D\right)$. Within the main loop process, both the physical evolution mechanism of PPO and the enhancement operations of the EES are predicated on basic linear vector operations; thus, their computational magnitude is equivalently $O\left(N\cdot D\right)$. Regarding the maintenance of the experience pool, since its capacity $N_{ep}$ is minimal and $N_{ep}\ll N$, its overhead is negligible in the asymptotic analysis.

In summary, incorporating the fitness evaluation costs incurred during each iteration, the total time complexity of EES-PPO, denoted as $T_{EESPPO}$, can be expressed as:

(23) $$T_{EESPPO}\approx O\left(T_{max}\cdot N\cdot \left(D+C_{obj}\right)\right)$$

The introduction of the EES does not alter the polynomial complexity order of the algorithm. $T_{EESPPO}$ maintains linear growth characteristics consistent with the original PPO algorithm. Consequently, it possesses the high efficiency required to address complex engineering problems, while simultaneously significantly enhancing global optimization accuracy.

### 3.4. Summary

In this chapter, we have constructed the EESPPO algorithm, which constitutes a systematic extension of the original PPO framework through the deep integration of dynamic experience pool maintenance, random guidance generation, and adaptive information fusion mechanisms. These improvement strategies function synergistically to not only effectively enrich the population’s evolutionary paths and break the limitations of guidance by a single optimal solution but also significantly enhance the algorithm’s capability to circumvent local optima within complex landscapes. Section 3.2 provides a detailed elucidation via pseudocode and flowcharts, visually demonstrating how the EES, acting as a probabilistic enhancement operator, is seamlessly coupled with the dual-track evolutionary cycle of PPO. The time complexity analysis in Section 3.3 further confirms that while introducing the historical experience guidance mechanism, EESPPO maintains the same linear complexity order as the standard PPO, incurring only a negligible increase in linear computational overhead.

In summary, EESPPO successfully establishes a general-purpose optimization framework that balances robustness with computational economy, providing powerful tool support for solving high-dimensional numerical problems and complex engineering applications.

## 4. Experimental Results and Analysis

This section provides a comprehensive evaluation of the proposed EESPPO algorithm against 12 existing advanced meta-heuristic algorithms, including PPO, HSO, SGA, PSO, FLO, DE, HO, WOA, KEO, GWO, FDB-AGSK, and IVYPSO. The experiments are divided into two parts: (1) Benchmark function evaluation using 29 CEC2017 functions; (2) Engineering application evaluation on four real-world engineering design problems.

### 4.1. Experimental Validation on CEC2017 Benchmark Functions

The proposed algorithm and comparative experiments were implemented in MATLAB R2024b. The testing environment was based on the Windows 11 operating system, utilizing a personal workstation equipped with an Intel(R) Core(TM) i5-14600KF CPU @ 3.50 GHz. To ensure a fair comparison, all algorithms were tested under the same settings. The population size and the dimension of the search space were both set to 30, while the termination condition for the algorithm was limited to 500 iterations. Each algorithm was executed for 30 independent runs on each benchmark function to eliminate stochastic bias. All algorithm-specific parameters were configured according to their original references or standard benchmark recommendations to ensure fairness and reproducibility. The detailed parameter settings for each algorithm are summarized in Table 1.

### 4.2. CEC2017 Benchmark Function Evaluation

To provide a comprehensive and unbiased evaluation of the performance differences between EESPPO and the competing algorithms in solving numerical optimization problems, four key statistical metrics were employed for quantitative analysis:

- **Best Value:** The minimum fitness value found by the algorithm across all independent runs.
- **Mean:** The arithmetic average of the results from all independent runs.
- **Standard Deviation:** The standard deviation of the results from all independent runs.
- **Average Rank:** Based on the results of the Friedman test, the performance ranking of all algorithms across each test set is quantified to derive the average ranking data.

The calculation methods for the relevant evaluation metrics (including Best, Mean, and Standard Deviation) are detailed in the following formulas:

(24) $$F_{best}=\underset{i=1}{\overset{M}{min}}f_{i},$$

(25) $$F_{mean}=\frac{1}{M}\sum_{i=1}^{M}f_{i},$$

(26) $$F_{std}=\sqrt{\frac{1}{M-1}\sum_{i=1}^{M}{\left(f_{i}-F_{mean}\right)}^{2}}$$

Furthermore, due to the stochastic nature of meta-heuristic algorithms, mean and standard deviation alone cannot fully determine whether the performance differences are statistically significant. Therefore, two non-parametric statistical tests were further adopted:

- **Friedman Rank Test:** Used to detect significant differences across multiple algorithms and provide an overall performance ranking.
- **Wilcoxon Signed-Rank Test:** Used to compare the performance difference between EESPPO and each competing algorithm at a 95% confidence level (significance level α = 0.05).

#### 4.2.1. Description of Benchmark Test Functions

To evaluate the performance of EESPPO across various types of numerical optimization problems, the internationally recognized IEEE CEC2017 benchmark suite was utilized. This dataset provides a rigorous and comprehensive standard for assessing an algorithm’s global search potential, local exploitation precision, and robustness. CEC2017 originally included 30 functions; however, F2 was officially deprecated due to instability in specific dimensions. This experiment utilized the remaining 29 functions (F1, F3–F30). The search range for all functions was set to [−100, 100].Based on the mathematical properties and the complexity of the fitness landscapes, these 29 functions are categorized into four classes:

- **Unimodal Functions (F1 & F3):** These functions have only one global optimum and no local minima, primarily used to test local exploitation capability and convergence speed.
- **Simple Multimodal Functions (F4–F10):** These contain numerous local optima that increase exponentially with dimensionality, designed to test global exploration and the ability to escape local optima.
- **Hybrid Functions (F11–F20):** Constructed by weighting, rotating, and shifting several basic functions (e.g., Rosenbrock, Rastrigin). they simulate complex real-world scenarios where different sub-problem features are coupled.
- **Composition Functions (F21–F30):** Formed by combining multiple sub-functions through complex weighting mechanisms. These are highly asymmetrical, non-separable, and multi-modal, representing the most challenging part of the suite for testing the balance between exploration and exploitation.

Detailed definitions, types, and theoretical optimal values of the CEC2017 test functions are shown in Table 2.

#### 4.2.2. Results and Analysis

To comprehensively evaluate the performance of the EESPPO algorithm, it was benchmarked against 12 representative algorithms—including PPO, SGA, HSO, PSO, FLO, DE, HO, WOA, KEO, GWO, IVYPSO, and FDB-AGSK—using the 29 functions from the CEC2017 benchmark test suite. Under identical experimental conditions (maximum iterations = 500, population size = 30, dimension = 30), each algorithm was executed independently 30 times for every test function.

As detailed in Table 3, among the 29 CEC2017 benchmark functions, EESPPO achieved the best performance across 24 functions. Regarding the unimodal functions (F1 and F3), EESPPO attained optimal mean values accompanied by minimal standard deviations. When addressing multimodal functions, which are characterized by numerous local optima, EESPPO maintained its dominance, securing the best results on six functions (F4, F6–F10).

Hybrid functions are designed to simulate complex real-world optimization landscapes. Here, EESPPO demonstrated superior performance across the majority of functions (F12–F20), being marginally surpassed only by PSO on F11. In the most challenging category of composition functions, EESPPO achieved optimal solutions on multiple functions, specifically F21–F24 and F28–F30. Although the original PPO exhibited certain competitiveness on F25–F27, the average ranking of EESPPO in this category remained superior to that of PPO and all other comparative algorithms. Across the entire test suite, EESPPO achieved an overall average rank of 1.21, underscoring its exceptional overall optimization capability.

This success is primarily attributed to the adaptive synergy among the Experience Scarcity, Experience Crossover, and Experience Sharing stages. This mechanism not only effectively maintains population diversity through the interaction of historical information but also significantly enhances convergence accuracy and robustness while balancing global exploration and local exploitation.

#### 4.2.3. Convergence Analysis

**Figure 4:**
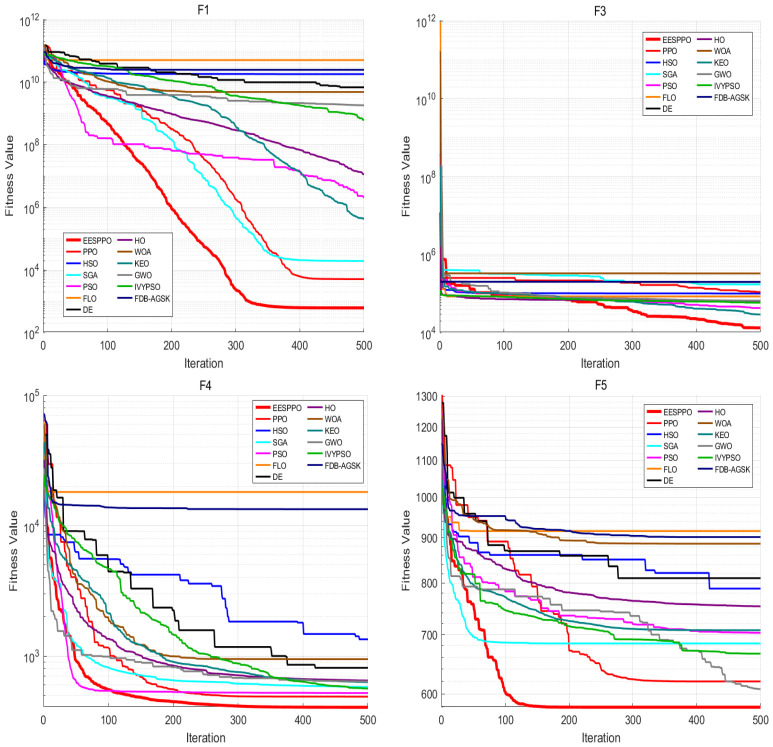

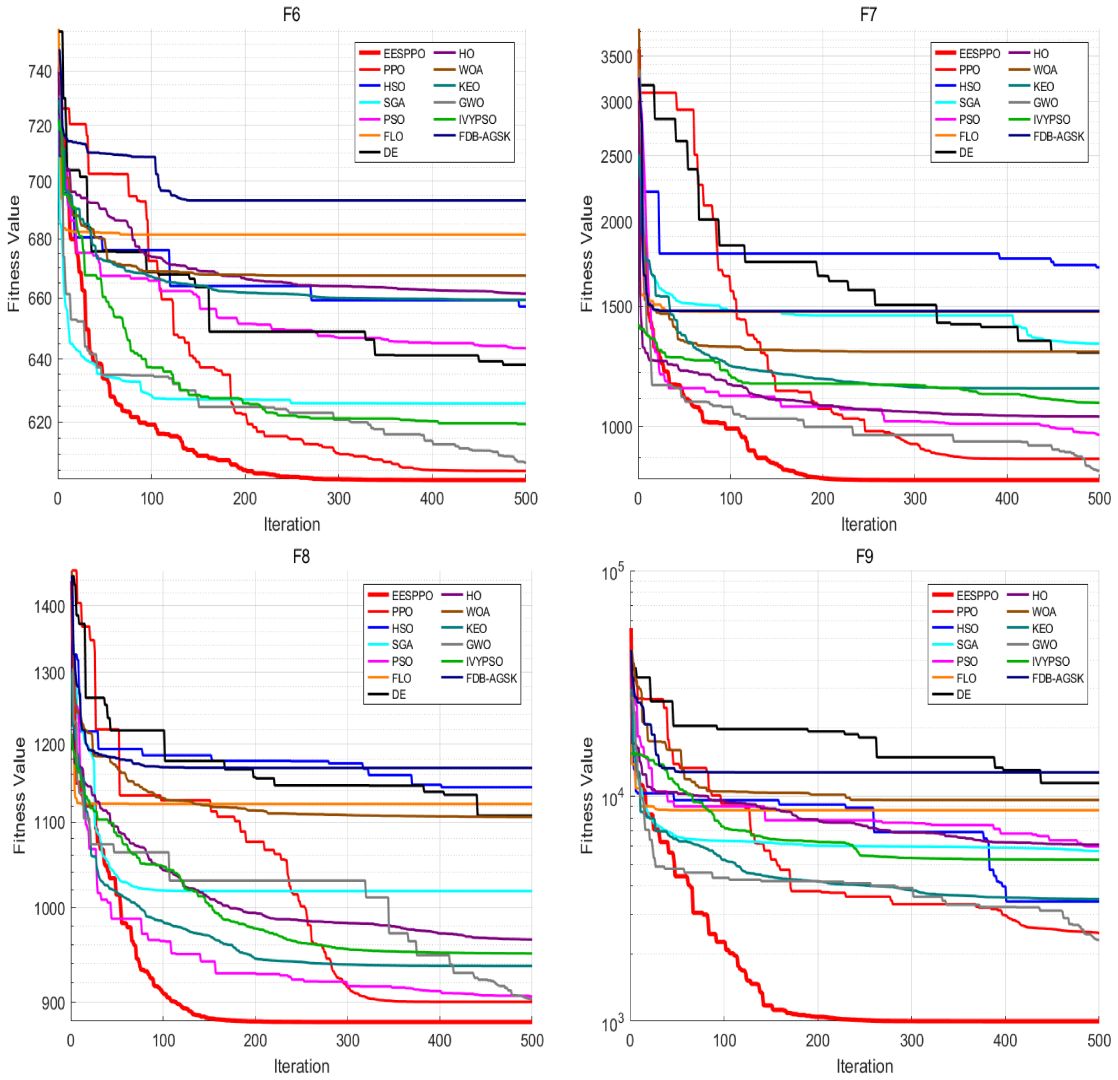

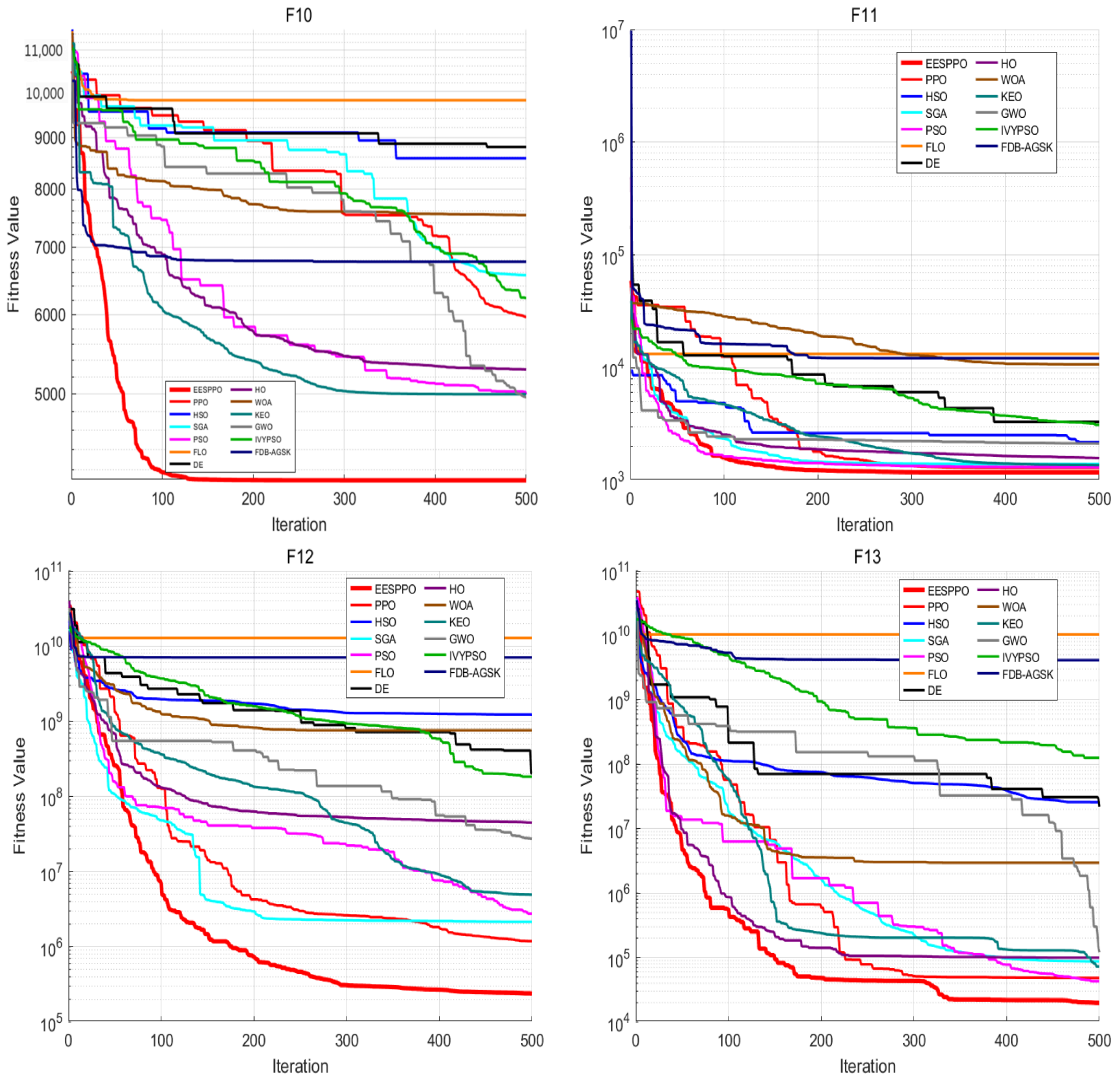

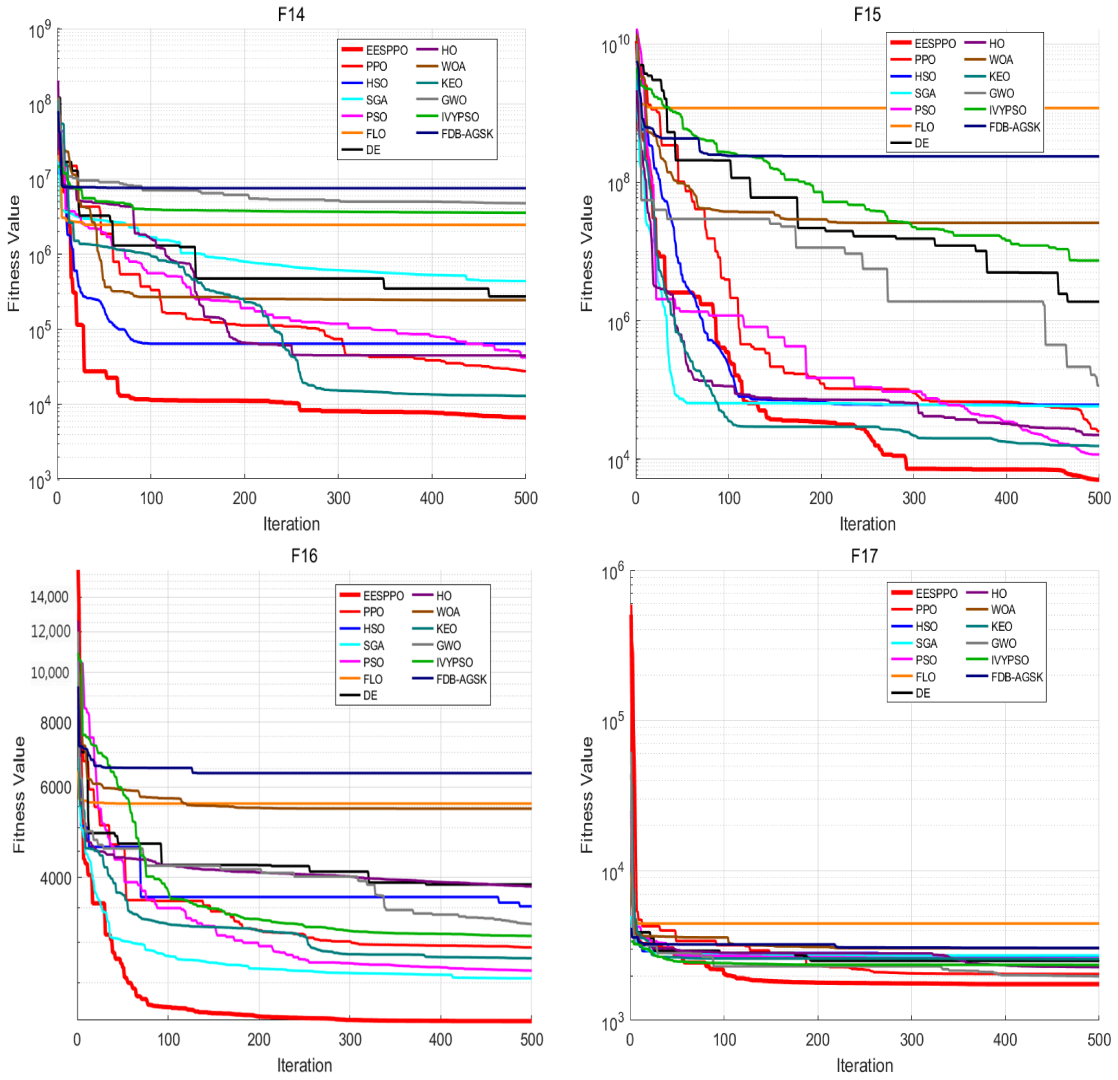

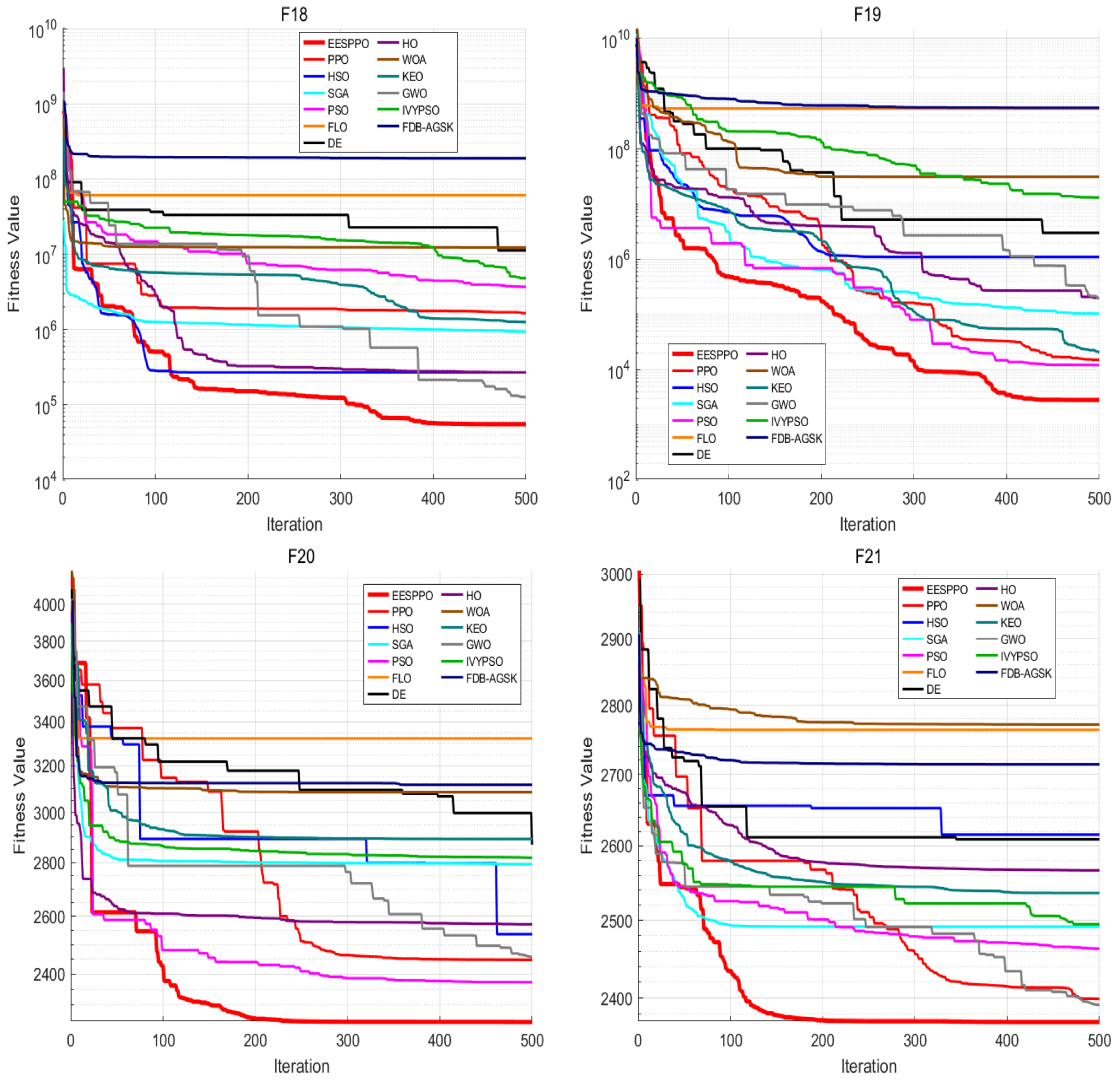

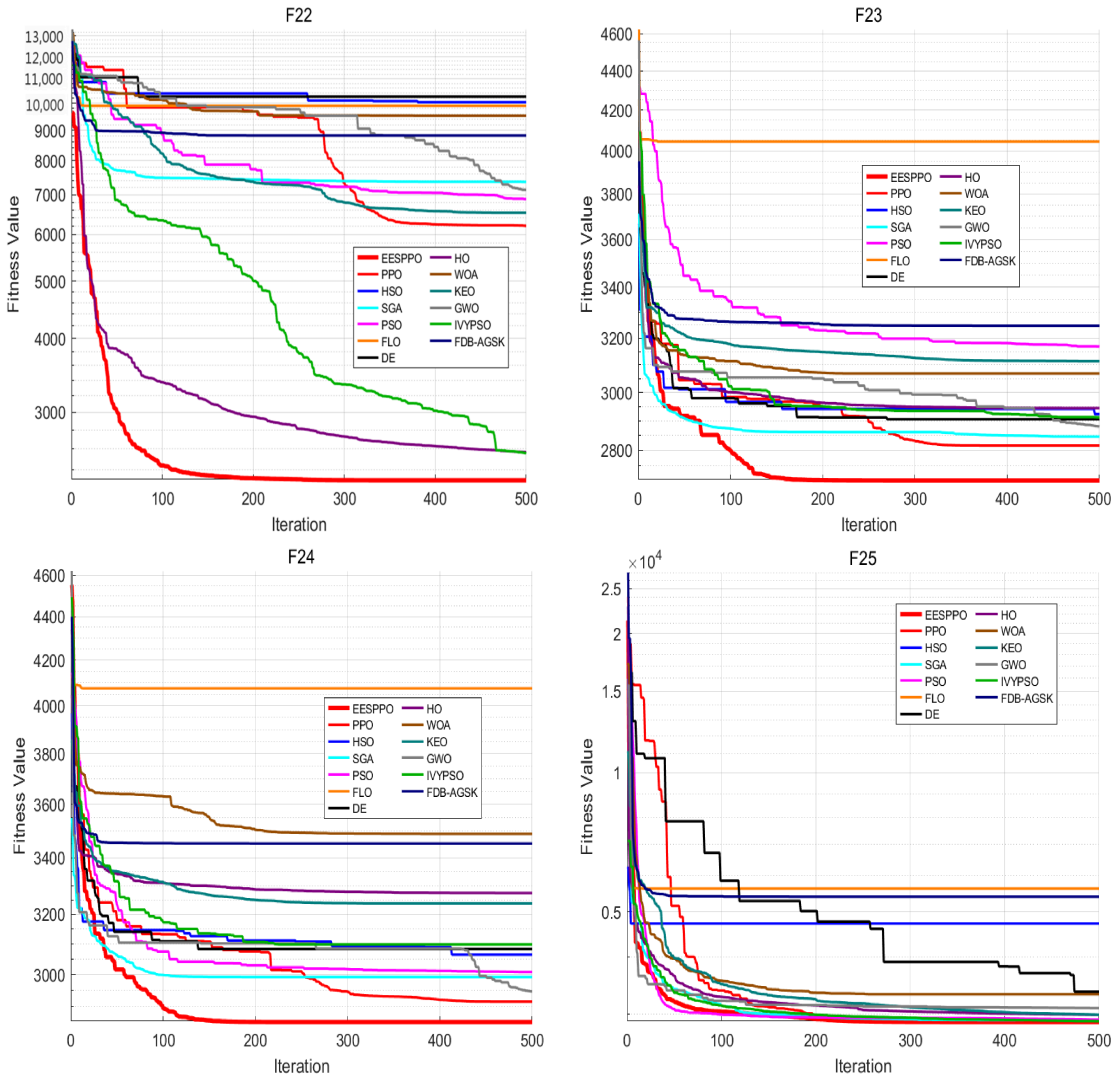

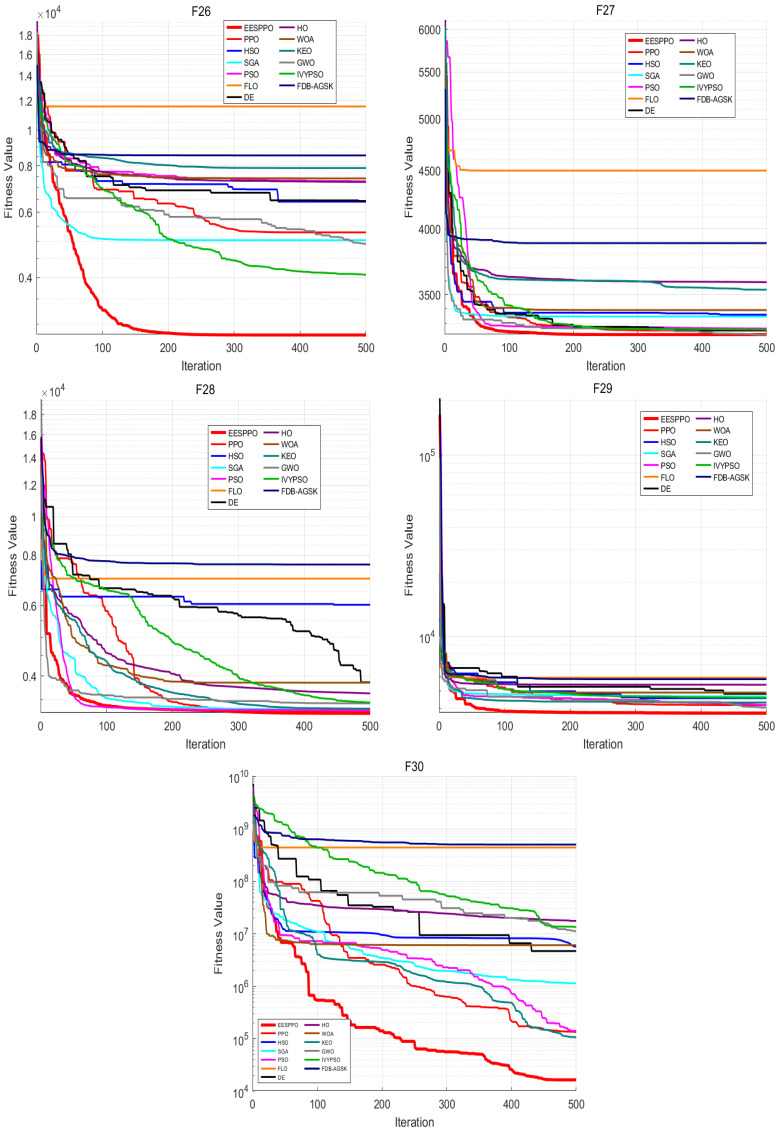
Convergence trends of the 13 comparative algorithms on the CEC2017 test suite.

Figure 4 illustrates the convergence curves of EESPPO benchmarked against other algorithms over 500 iterations on the CEC2017 benchmark functions. Among the 29 test functions, EESPPO demonstrates consistent, rapid convergence and superior exploitation efficiency across the vast majority of cases. In particular, it exhibits remarkably superior optimization capabilities on specific functions—including F1, F5–F10, F12, F14, F16, F18–F24, F26, and F30—with an overall performance that significantly surpasses the other 12 state-of-the-art algorithms. These results confirm the excellent balance EESPPO achieves between convergence speed and solution accuracy, further highlighting its exceptional robustness and comprehensive computational advantages when dealing with high-dimensional complex landscapes.

#### 4.2.4. Results of Friedman Ranking and Wilcoxon Signed-Rank Analysis

To further validate whether the performance advantages of EESPPO observed in the aforementioned experiments are statistically significant rather than merely attributable to random probability, this section employs two non-parametric statistical methods for in-depth analysis: the Friedman ranking test and the Wilcoxon signed-rank test.

**Figure 5:**
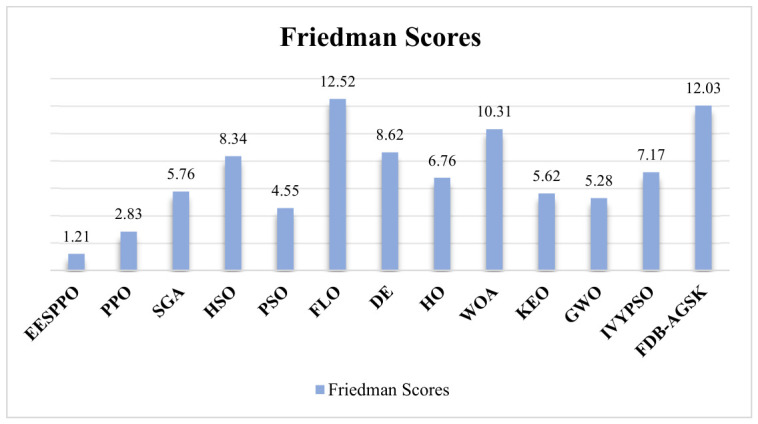
Friedman ranking scores of 13 algorithms.

Figure 5 presents the Friedman ranking results for the 13 algorithms across the 29 benchmark functions. In terms of specific ranking performance, EESPPO demonstrated superior performance, achieving the best overall status with a mean rank of 1.21. This result is not only significantly superior to the second-ranked original PPO (average rank 2.83) and the third-ranked PSO (average rank 4.55), but it also establishes a substantial gap between EESPPO and lower-ranked algorithms such as FLO and FDB-AGSK. This fully demonstrates EESPPO’s strongest comprehensive adaptability and robustness when handling diverse types of benchmark functions.

To further analyze the significance of the differences between the proposed algorithm and the other 12 comparative algorithms in pairwise comparisons, we performed a Wilcoxon signed-rank test at a 5% significance level (α = 0.05). Table 4 details the Wilcoxon test results between EESPPO and each competitor. The statistical data indicate that EESPPO demonstrated overwhelming statistical dominance in all pairwise comparisons. All calculated *p*-values are far below the threshold of 0.05 (with magnitudes ranging between 10^−5^ and 10^−9^). This implies that, with a 95% confidence level, the optimization performance of EESPPO is significantly superior to that of all selected competitors.

The aforementioned statistical analyses robustly confirm that the performance advantage of EESPPO does not stem from random chance but possesses clear statistical significance. This fully verifies the high reliability and exceptional generalization capability of the algorithm when addressing different problems.

In summary, the dual validation provided by the Friedman ranking and the Wilcoxon signed-rank test consistently indicates that EESPPO not only ranks first in average performance but also constitutes a statistically significant improvement over current mainstream and cutting-edge optimization algorithms. This confirms the high robustness, high precision, and superior optimization capabilities of the proposed EESPPO algorithm.

### 4.3. Engineering Optimization

To further verify the robustness and stability of EES-PPO in handling various real-world engineering tasks with complex constraints, four classic mechanical engineering design problems were selected for benchmarking: the tension/compression spring design, the gas transmission compressor design, the welded beam design, and the speed reducer design. These problems typically involve non-linear objective functions, mixed variable types, and intricate equality or inequality constraints; this poses a severe test of the stability and robustness of the algorithm.

For all problems, the population size and maximum iteration were set to the same parameters used in benchmark testing (30 and 500, respectively). Each algorithm was executed 30 independent runs, and results were evaluated by Best, Mean, Std, Average Rank, and Average Computation Time (ACT).

#### 4.3.1. Translation of Engineering Problems and Constraint Handling

Before deploying the EESPPO algorithm to solve specific real-world mechanical designs, it is essential to establish a standardized framework that translates these practical physical constraints into a mathematical paradigm computable by the algorithm.

In the EESPPO framework, each individual (search agent) within the population represents a candidate design scheme. For an engineering problem with D independent design variables, the position vector of the i-th individual, $X_{i}=\left[x_{i,1},x_{i,2},\ldots ,x_{i,D}\right]$, encodes a specific combination of physical parameters (e.g., structural dimensions, thicknesses, or material properties). The search space boundaries $\left[L,U\right]$ strictly correspond to the permissible physical manufacturing ranges of these parameters.

Furthermore, unlike the unconstrained CEC2017 benchmark functions, real-world engineering problems are characterized by rigorous inequality and equality constraints ensuring structural safety and functional feasibility. Since EESPPO is inherently an unconstrained optimizer, we employ the Static Penalty Function Method to seamlessly integrate the physical constraints into the objective evaluation. The generalized constrained optimization problem is formulated as:

Minimize: $f\left(X\right)$
subject to:

(27) $$g_{j}\left(X\right)\leq 0,\;j=1,2,\ldots ,p$$

(28) $$h_{k}\left(X\right)=0,\;k=1,2,\ldots ,q$$

where $f\left(X\right)$ is the original physical objective (e.g., manufacturing cost or structural weight), while $g_{j}\left(X\right)$ and $h_{k}\left(X\right)$ denote the inequality and equality constraints, respectively. To process this within EESPPO, the problem is mapped to an unconstrained fitness function $F\left(X\right)$ using severe penalty terms:

(29) $$F\left(X\right)=f\left(X\right)+\lambda_{1}\sum_{j=1}^{p}max{\left(0,g_{j}\left(X\right)\right)}^{2}+\lambda_{2}\sum_{k=1}^{q}{\left|h_{k}\left(X\right)\right|}^{2}$$

where $\lambda_{1}$ and $\lambda_{2}$ are massive penalty coefficients (typically set to 10^10^ or higher in this study). During the evolutionary process, any candidate vector $X_{i}$ that violates a physical constraint will trigger the penalty terms, resulting in an astronomically high fitness value $F\left(X_{i}\right)$. Driven by the Experience Exchange Strategy (EES)—particularly the greedy elimination in the ESC stage—these infeasible solutions are rapidly discarded by the swarm. This constraint-handling mapping ensures that the final optimal solution generated by EESPPO is not only mathematically optimal but also strictly compliant with physical and manufacturing regulations.

#### 4.3.2. Tension/Compression Spring Design

**Figure 6:**
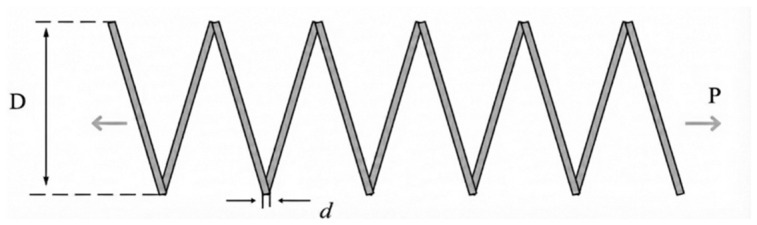
Tension/compression string design problem.

The schematic diagram of the tension/compression spring design problem is visualized in Figure 6 [46]. The core objective of this problem is to minimize the overall weight of the spring by optimizing its geometric parameters, subject to strict physical constraints regarding shear stress, surge frequency, and deflection.

This design problem involves three continuous decision variables: wire diameter d (a_1_), mean coil diameter D (a_2_), and the number of active coils N (a_3_). The mathematical formulation of this problem is characterized by high non-linearity and complex coupling relationships among constraints, posing a severe challenge to the search capability of optimization algorithms within a narrow feasible region. The mathematical description is presented in Equation (30):

Minimize:

(30) $$f\left(a\right)=\left(a_{3}+2\right)a_{2}a_{1}^{2}$$

subject to:

$$g_{1}\left(a\right)=1-\frac{a_{2}^{3}a_{3}}{71785a_{1}^{4}}\leq 0,$$

$$g_{2}\left(a\right)=\frac{4a_{2}^{2}-a_{1}a_{2}}{12566\left(a_{2}a_{1}^{3}-a_{1}^{4}\right)}+\frac{1}{5108a_{1}^{2}}-1\leq 0,$$

$$g_{3}\left(a\right)=1-\frac{140.45a_{1}}{a_{2}^{2}a_{3}}\leq 0,$$

$$g_{4}\left(a\right)=\frac{a_{1}+a_{2}}{1.5}-1\leq 0.$$

where:

$$0.05\leq a_{1}\leq 2.00,\;0.25\leq a_{2}\leq 1.30,\;2.00\leq a_{3}\leq 15.00$$

Table 5 details the optimal design solutions and computational efficiency achieved by each algorithm over 30 independent runs. EESPPO accurately identified the global optimal design variables [d, D, N] = [0.0517, 0.3574, 11.2472], which satisfy all constraints. The corresponding minimum weight is 0.0127, matching the theoretical optimum. Furthermore, its Average Computing Time (ACT) is 0.1726 s, which is comparable to that of the original PPO (0.1662 s), demonstrating a favorable balance between algorithmic performance enhancement and computational cost. Table 6 summarizes the robustness metrics for each algorithm. The mean fitness, median fitness, best value, and worst value for EESPPO all stabilized at 0.0127, achieving perfect convergence with zero fluctuation. Compared with other optimization algorithms, EESPPO exhibits exceptional stability, robustness, and high engineering reliability when addressing complex and strongly constrained problems.

#### 4.3.3. Gas Transmission Compressor Design Problem

**Figure 7:**
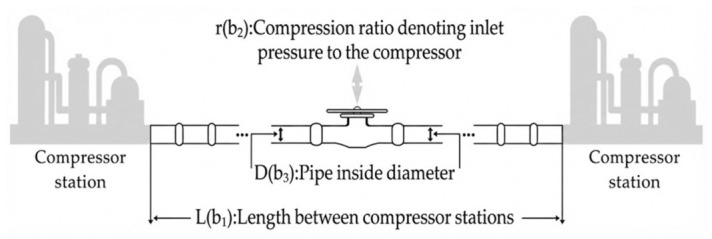
Conceptual diagram of the Gas Transmission Compressor Design Problem.

The schematic diagram of the gas transmission compressor design problem is visualized in Figure 7 [47]. The primary objective of this problem is to minimize the total system cost, which encompasses both the pipeline construction cost and the compressor operating cost. This is achieved by optimizing the key parameters of the pipeline and compressor, subject to the strict constraint that the gas transmission process must satisfy specific dynamic requirements.

This system comprises three core decision variables: the length of the pipe segment L (b_1_), the compression ratio r(b_2_), and the pipe diameter parameter D(b_3_). The problem involves complex exponential operations and variable coupling, posing a significant challenge to the global optimization capability of algorithms within multi-modal landscapes. The mathematical formulation is presented in Equation (31):

Minimize:

(31) $$\begin{array}{ll} f(b)= & 8.61\times 10^{5}{{{b_{1}}^{\frac{1}{2}}b_{2}b}_{3}}^{-\frac{2}{3}}{\left({x_{2}}^{2}-1\right)}^{-\frac{1}{2}}\\ & -765.43\times 10^{8}{b_{1}}^{-1}+3.69\times 10^{4}b_{3}\\ & +7.72\times 10^{8}{b_{1}}^{-1}{b_{2}}^{0.219}, \end{array}$$

subject to:

$$g_{1}\left(b\right)=b_{3}^{-2}+b_{2}^{2}-1\leq 0$$

where

$$10\leq b_{1}\leq 55,\;1.1\leq b_{2}\leq 2.0,\;10\leq b_{3}\leq 40$$

Table 7 and Table 8 present the detailed experimental results. The experiments demonstrate that EESPPO successfully identified the global optimal design parameters [L, r, D] = [24.4690, 1.1587, 20.0000] that satisfy all constraints. The corresponding minimum cost is 1,677,759.2755. Across 30 independent runs, the mean, median, and worst values all stabilized at this theoretical optimum, with a standard deviation of 0, indicating perfect convergence with zero fluctuation. Furthermore, the Average Computing Time (ACT) for EESPPO was recorded at a mere 0.2947 s, which is lower than that of all other comparative algorithms. This proves that while maintaining high-precision convergence, the algorithm keeps computational costs within a highly practical and reasonable range, effectively balancing engineering reliability with real-time performance requirements.

#### 4.3.4. Welded Beam Design Problem

**Figure 8:**
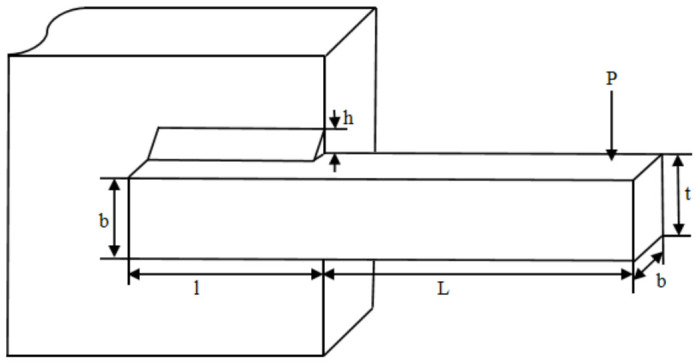
Schematic diagram of the welded beam design problem.

The schematic diagram of the welded beam design problem is visualized in Figure 8 [18]. The primary objective of this problem is to minimize the total fabrication cost—encompassing both welding labor and raw material costs—by optimizing the geometric dimension parameters. This optimization process is subject to the strict satisfaction of seven physical constraints, including shear stress, bending stress, buckling load on the bar, and end deflection.

This design involves four continuous decision variables: weld thickness h(c_1_), length of the attached bar l(c_2_), height of the bar t(c_3_), and thickness of the bar b(c_4_). The objective function of the mathematical model comprises two non-linear cost components, aiming to strike an optimal balance between material utilization and structural safety. Since the constraints involve intricate mechanical formulas and geometric boundary limitations, strong coupling relationships exist among the variables. This poses a rigorous challenge to the algorithm’s precise search capability within a restricted feasible space. The mathematical formulation is described in Equation (32):

Minimize:

(32) $$f\left(c\right)=1.10471c_{1}^{2}c_{2}+0.04811c_{3}c_{4}\left(14.0+c_{2}\right)$$

subject to:

$$g_{1}\left(c\right)=\tau \left(c\right)-\tau_{max}\leq 0,$$

$$g_{2}\left(c\right)=\sigma \left(c\right)-\sigma_{max}\leq 0,$$

$$g_{3}\left(c\right)=c_{1}-c_{4}\leq 0,$$

$$g_{4}\left(c\right)=0.10471c_{1}^{2}+0.04811c_{3}c_{4}\left(14.0+c_{2}\right)-5.0\leq 0,$$

$$g_{5}\left(c\right)=0.125-c_{1}\leq 0,$$

$$g_{6}\left(c\right)=\delta \left(c\right)-\delta_{max}\leq 0,$$

$$g_{7}\left(c\right)=P-P_{c}\left(c\right)\leq 0$$

where:

$$0.1\leq c_{1},c_{4}\leq 2.0,$$

$$0.1\leq c_{2},c_{3}\leq 10.0$$

Table 9 and Table 10 present the detailed experimental results. In terms of optimization accuracy and robustness, EESPPO successfully identified the global optimal design parameters [h, l, t, b] = [0.1988, 3.3373, 9.1918, 0.1988] that satisfy all seven constraints. The corresponding minimum fabrication cost is 1.6702. Across 30 independent runs, the mean, median, and worst values of the algorithm strictly converged to this theoretical optimum, with a standard deviation of 0.0000, achieving perfect convergence with zero fluctuation. Furthermore, the Average Computing Time (ACT) remained consistently within a reasonable range. Compared with other optimization algorithms, EESPPO exhibited superior comprehensive performance in solving this problem, further validating its exceptional stability and robustness.

#### 4.3.5. Speed Reducer Design Problem

**Figure 9:**
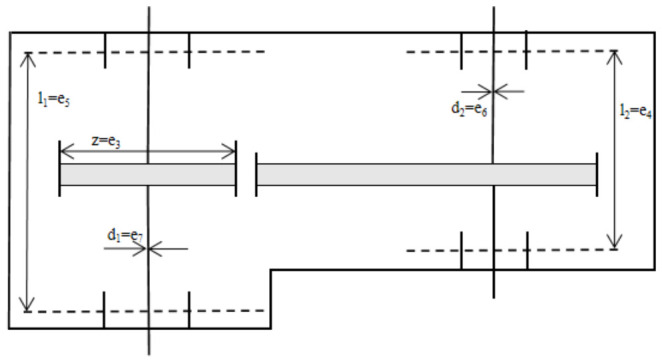
Schematic diagram of weight minimization of a speed reducer problem(dashed lines indicate the locations of the bearings and housing supports).

The schematic diagram of the speed reducer design problem is visualized in Figure 9 [48]. The primary objective of this problem is to minimize the total weight of the speed reducer, subject to the strict satisfaction of 11 complex geometric and mechanical constraints. These constraints specifically involve the surface stress on gear teeth, bending stress on gear teeth, transverse deflection of shafts, and stresses in shafts.

This design problem optimizes seven continuous variables: the face width b(e_1_), the module of teeth m (e_2_), and number of teeth on the pinion z(e_3_), while the remaining variables represent the bearing spans(l_1_(e_4_), l_2_(e_5_))and diameters (d_1_(e_6_), d_2_(e_7_)) of the first and second shafts. The objective function of the mathematical model is composed of the weights of the gears, shafts, and bearing structures. It is characterized by high non-linearity and complex coupling relationships among constraints, which drastically compress the feasible region space. The complete mathematical description is presented in Equation (33):

Minimize:

(33) $$\begin{array}{ll} f\left(e\right) & =0.7854e_{1}e_{2}^{2}\left(3.3333e_{3}^{2}+14.9334e_{3}-43.0934\right)\\ & -1.508e_{1}\left(e_{6}^{2}+e_{7}^{2}\right)+7.4777\left(e_{6}^{3}+e_{7}^{3}\right)+0.7854\left(e_{4}e_{6}^{2}+e_{5}e_{7}^{2}\right) \end{array}$$

subject to:

$$g_{1}\left(e\right)=\frac{27}{e_{1}e_{2}^{2}e_{3}}-1\leq 0,$$

$$g_{2}\left(e\right)=\frac{397.5}{e_{1}e_{2}^{2}e_{3}^{2}}-1\leq 0,$$

$$g_{3}\left(e\right)=\frac{1.93e_{4}^{3}}{e_{2}e_{3}e_{6}^{4}}-1\leq 0,$$

$$g_{4}\left(e\right)=\frac{1.93e_{5}^{3}}{e_{2}e_{3}e_{7}^{4}}-1\leq 0,$$

$$g_{5}\left(e\right)=\frac{1.0}{110e_{6}^{3}}\sqrt{{\left(\frac{745e_{4}}{e_{2}e_{3}}\right)}^{2}+16.9\times 10^{6}}-1\leq 0,$$

$$g_{6}\left(e\right)=\frac{1.0}{85e_{7}^{3}}\sqrt{{\left(\frac{745e_{5}}{e_{2}e_{3}}\right)}^{2}+157.5\times 10^{6}}-1\leq 0,$$

$$g_{7}\left(e\right)=\frac{e_{2}e_{3}}{40}-1\leq 0,$$

$$g_{8}\left(e\right)=\frac{5e_{2}}{e_{1}}-1\leq 0,$$

$$g_{9}\left(e\right)=\frac{e_{1}}{12e_{2}}-1\leq 0,$$

$$g_{10}\left(e\right)=\frac{1.5e_{6}+1.9}{e_{4}}-1\leq 0,$$

$$g_{11}\left(e\right)=\frac{1.1e_{7}+1.9}{e_{5}}-1\leq 0,$$

where

$$2.6\leq e_{1}\leq 3.6,\;0.7\leq e_{2}\leq 0.8,\;17\leq e_{3}\leq 28,\;7.3\leq e_{4}\leq 8.3,$$

$$7.3\leq e_{5}\leq 8.3,\;2.9\leq e_{6}\leq 3.9,\;5.0\leq e_{7}\leq 5.5$$

Table 11 and Table 12 present the detailed experimental results. In terms of optimization outcomes, EESPPO successfully pinpointed a set of global optimal design parameters [b, m, z, l_1_, l_2_, d_1_, d_2_] = [3.4990, 0.7000, 17.0000, 7.3000, 7.7152, 3.3505, 5.2867] that satisfy all 11 constraints. The corresponding minimum speed reducer weight is 2,994.2343.Across 30 independent runs, all experiments yielded the identical objective value of 2,994.2343, achieving perfect convergence with zero deviation, while also demonstrating a highly efficient Average Computing Time (ACT). The experiments prove that EESPPO maintains an efficient balance between exploration and exploitation even within an extremely complex design space, possessing both high precision and robustness in solving this problem.

## 5. Conclusions

This paper presents an enhanced swarm intelligence algorithm based on the social behavior of *Philoponella prominens*, termed EESPPO, designed to address the complex challenges associated with high-dimensional nonlinearity and rigorous constraints prevalent in engineering optimization. Grounded in the cooperative predatory behavior of spiders, the algorithm incorporates an innovative hierarchical progressive experience exchange strategy to reshape the population’s search dynamics. Specifically, during the Experience Scarcity (ESC) stage, a dynamic experience pool is constructed to retain superior historical information during the evolutionary process. In the Experience Crossover (ECR) stage, a perturbation mechanism utilizing random guidance vectors is employed to enhance the algorithm’s ability to escape local optima. Finally, in the Experience Sharing (ESH) stage, an adaptive fusion update strategy is applied to eliminate information isolation among individuals and foster global collaboration.

Comprehensive experiments conducted on the 29 CEC2017 benchmark functions and four challenging engineering design problems (tension/compression spring design, gas transmission compressor design, welded beam design, and speed reducer weight minimization) demonstrate that EESPPO exhibits global optimization capabilities and convergence speeds superior to those of 12 mainstream algorithms. Particularly regarding constraint handling in engineering problems, EESPPO precisely converged to the theoretical optimum in all independent runs, achieving perfect convergence with zero fluctuation. This underscores its extraordinary stability and robustness, proving that EESPPO possesses both powerful capabilities for solving complex problems and high practical engineering value.

Future research will focus on extending the EESPPO framework to multi-objective optimization scenarios, thereby addressing more intricate engineering design challenges. Furthermore, we aim to apply the algorithm to solve complex real-world optimization problems, such as multi-threshold image segmentation and UAV path planning.

## Figures and Tables

**Table 1:** Parameter settings of the 13 algorithms.

Algorithms	Parameter	Algorithms	Parameter
ALL	Max iteration = 500, Agents = 30, Runs = 30, dim = 30	GWO	$a=2-2\left(t/T\right),r_{1},r_{2}\in \left[0,1\right]$
EESPPO	$\beta =1.5,\sigma_{coef}=\left(1-t/T\right)+0.5,$ $S_{pool}=min\left(20,N\right),P_{EES}=0.3,\alpha =0.5+0.3\left(1-t/T\right)$	WOA	1=r2=rand,r C=2∗r2,b=1
PPO	$\beta =1.5,\sigma_{coef}=\left(1-t/T\right)+0.5$	KEO	$GS=0.05N,ET=0.5,\beta_{1}=1,$ $\theta_{max}=30^{\circ },x_{0}=0.7,P_{safe}=0.75$
HSO	$\alpha =3,T_{0}=10000,cr=0.995,$ $mr=0.5-0.4\left(t/T\right),ms=0.3-0.2\left(t/T\right)$	DE	F=0.9,CR=0.5
HO	$I_{1},I_{2}\in \left[1,2\right],b\in \left[2,4\right],c\in \left[1,1.5\right],$ $d\in \left[2,3\right],l\in \left[-2\pi ,2\pi \right],\beta =1.5$	PSO	Vmax=6,wMax=0.9,wMin=0.6, c1=2,c2=2
SGA	$L=0.5,h=6.625\times 10^{-34},b=1-{\left(t/T\right)}^{0.2},$ P=0.03,β=1.5	FDB-AGSK	a1=2−t∗(2/Max_iter), a2=1.5−t∗(1.5/Max_iter)
FLO	$I=round\left(1+rand\right),P_{switch}=0.5$	IVYPSO	$w=0.7,c_{1}=1.5,c_{2}=1.5,\beta_{1}\in \left[1,1.5\right]$

**Table 2:** CEC2017 test functions F1, F3–F30.

Category	Function ID	Function Name	Search Range	Global Optimum
Unimodal	F1	Shifted and Rotated Bent Cigar	[−100, 100]^Dim	100
Unimodal	F3	Shifted and Rotated Zakharov	[−100, 100]^Dim	300
Simple Multimodal	F4	Shifted and Rotated Rosenbrock	[−100, 100]^Dim	400
Simple Multimodal	F5	Shifted and Rotated Rastrigin	[−100, 100]^Dim	500
Simple Multimodal	F6	Shifted and Rotated Schaffer’s F7	[−100, 100]^Dim	600
Simple Multimodal	F7	Shifted and Rotated Ackley	[−100, 100]^Dim	700
Simple Multimodal	F8	Shifted and Rotated Griewank	[−100, 100]^Dim	800
Simple Multimodal	F9	Shifted and Rotated Weierstrass	[−100, 100]^Dim	900
Simple Multimodal	F10	Shifted and Rotated Schwefel	[−100, 100]^Dim	1000
Hybrid Functions	F11	Hybrid Function 1 (Schaffer F7 + Rosenbrock)	[−100, 100]^Dim	1100
Hybrid Functions	F12	Hybrid Function 2 (Rastrigin + Griewank + Rosenbrock)	[−100, 100]^Dim	1200
Hybrid Functions	F13	Hybrid Function 3 (Ackley + Rastrigin + Bent Cigar)	[−100, 100]^Dim	1300
Hybrid Functions	F14	Hybrid Function 4 (Zakharov + Rastrigin + Schaffer F7 + Rosenbrock)	[−100, 100]^Dim	1400
Hybrid Functions	F15	Hybrid Function 5 (Ackley + Rastrigin + Griewank + Bent Cigar + Schwefel)	[−100, 100]^Dim	1500
Hybrid Functions	F16	Composition Function 1 (5 Base Functions)	[−100, 100]^Dim	1600
Hybrid Functions	F17	Composition Function 2 (Rastrigin + Griewank + Ackley + Schwefel + Rosenbrock)	[−100, 100]^Dim	1700
Hybrid Functions	F18	Composition Function 3 (5 Hybrid Functions)	[−100, 100]^Dim	1800
Hybrid Functions	F19	Composition Function 4 (6 Base Functions)	[−100, 100]^Dim	1900
Hybrid Functions	F20	Composition Function 5 (Rastrigin + Ackley + Griewank + Schwefel + Zakharov)	[−100, 100]^Dim	2000
Composition Functions	F21	Composition Function 6 (Weierstrass + Rosenbrock + Ackley + Griewank + Rastrigin)	[−100, 100]^Dim	2100
Composition Functions	F22	Composition Function 7 (6 Sub-functions)	[−100, 100]^Dim	2200
Composition Functions	F23	Composition Function 8 (5 Sub-functions)	[−100, 100]^Dim	2300
Composition Functions	F24	Composition Function 9 (5 Sub-functions, Rotated)	[−100, 100]^Dim	2400
Composition Functions	F25	Composition Function 10 (6 Sub-functions, Layered)	[−100, 100]^Dim	2500
Composition Functions	F26	Composition Function 11 (Hybrid + Rastrigin + Rosenbrock)	[−100, 100]^Dim	2600
Composition Functions	F27	Composition Function 12 (6 Hybrid Sub-functions)	[−100, 100]^Dim	2700
Composition Functions	F28	Composition Function 13 (Hybrid + Griewank + Ackley)	[−100, 100]^Dim	2800
Composition Functions	F29	Composition Function 14 (Hybrid + Schwefel + Rosenbrock)	[−100, 100]^Dim	2900
Composition Functions	F30	Composition Function 15 (Hybrid + Bent Cigar + Rastrigin)	[−100, 100]^Dim	3000

**Table 3:** The statistical results of the 13 algorithms on the 29 benchmark functions, including best values, mean values, standard deviations, and the mean ranks derived from average fitness.

Function/Metric	EESPPO	PPO	SGA	HSO	PSO	FLO	DE	HO	WOA	KEO	GWO	IVYPSO	FDB-AGSK
F1_Best	100.0256	106.2591	116.1924	7,943,287,748	702,890.9567	37,178,612,402	3,650,074,920	3,674,407.44	2,149,304,038	89,482.7651	691,071,123.9	329,628,736.9	32,290,643,145
F1_Mean	1907.4594	4007.4984	5343.3913	12,971,995,099	2,912,550.87	56,571,728,505	5,893,744,593	147,394,822.6	5,541,835,568	819,183.3508	3,398,751,343	2,433,869,790	49,947,690,279
F1_Std	2276.9127	5169.579	6428.7786	3,173,390,388	1,378,995.624	7,273,672,849	1,042,789,577	230,137,932.9	1,749,221,925	790,146.1655	2,460,165,130	2,531,691,767	7,857,643,752
F1_Rank	1	2	3	11	5	13	10	6	9	4	8	7	12
F3_Best	13,127.6981	42,520.9533	38,116.6467	46,441.6689	17,034.3388	72,612.936	187,624.3217	39,656.7857	127,581.3754	8764.5307	41,383.9364	58,206.5651	136,278.5501
F3_Mean	26,471.3659	78,934.3717	105,263.6442	82,284.8174	41,472.534	89,929.0583	252,773.5725	51,327.2761	260,839.4206	29,271.9623	64,382.1755	74,804.1876	251,616.1155
F3_Std	9956.6883	23,319.3696	32,603.3837	18,168.0277	14,005.9913	5418.9106	26,206.1327	6598.3327	76,029.5477	13,307.4016	14,484.6954	9467.7075	72,555.5423
F3_Rank	1	7	10	8	3	9	12	4	13	2	5	6	11
F4_Best	468.942	471.7951	448.8651	1193.1605	468.3443	9479.2881	770.8455	481.8503	891.5466	473.4063	521.0569	546.7332	6302.9039
F4_Mean	499.2995	505.4899	538.7365	1667.3195	499.6734	15,675.9383	942.9647	605.066	1392.958	544.2308	605.8151	939.4519	12,777.9846
F4_Std	19.1064	24.7138	53.4128	533.9326	20.935	2989.1293	127.7684	62.2368	415.4761	39.994	53.2138	505.0194	3286.0391
F4_Rank	1	3	4	11	2	13	9	6	10	5	7	8	12
F5_Best	544.7731	555.0481	573.6267	763.2649	603.4575	837.4081	756.3705	673.4608	753.4053	597.5122	568.4639	644.1718	884.6853
F5_Mean	586.6938	582.654	695.2295	800.5019	685.0744	925.8958	794.1535	726.8677	855.1786	703.5488	626.601	717.4046	945.2751
F5_Std	19.363	20.2095	52.9989	20.3414	34.9287	34.2568	18.1161	34.1205	56.6506	44.7693	30.9242	36.9299	35.2792
F5_Rank	2	1	5	10	4	12	9	8	11	6	3	7	13
F6_Best	602.937	603.2691	622.1903	643.233	637.2438	681.1671	623.9594	647.3642	662.409	636.9143	604.4901	613.2	669.0703
F6_Mean	608.8433	610.8923	643.9594	656.9744	649.0859	693.469	634.6388	659.1535	678.5637	649.4527	612.7651	630.1172	696.2329
F6_Std	5.1759	4.6033	10.0218	5.7822	7.0123	6.6811	3.4626	5.7499	9.9338	6.8516	4.4567	11.3666	13.3758
F6_Rank	1	2	6	9	7	12	5	10	11	8	3	4	13
F7_Best	789.5328	799.5294	1100.9002	1421.716	848.4781	1300.5829	1125.7762	1005.4821	1188.0207	975.0735	848.8	1103.0296	1300.6055
F7_Mean	838.0408	862.7625	1317.5288	1531.794	917.4438	1437.5269	1254.871	1131.8307	1299.6897	1187.1478	903.7384	1265.3054	1450.7817
F7_Std	27.8508	41.1105	113.2973	65.1937	33.1538	50.9702	51.9727	57.9371	67.5719	114.1688	42.8669	69.6623	60.2536
F7_Rank	1	2	10	13	4	11	7	5	9	6	3	8	12
F8_Best	837.8084	838.8036	898.9736	1091.1355	901.4126	1113.087	1050.4395	902.8141	995.1038	884.5733	862.1534	892.4755	1104.8824
F8_Mean	881.3874	894.6221	992.9062	1119.2332	935.4908	1153.9445	1098.9576	959.6607	1069.0041	953.9311	895.6583	944.9481	1155.4381
F8_Std	18.9634	33.0438	48.7957	11.651	22.55041	26.1444	19.1392	23.0251	40.6141	29.3968	24.8938	28.525	33.9411
F8_Rank	1	2	8	11	4	12	10	7	9	6	3	5	13
F9_Best	945.0155	1038.8167	3870.7441	2903.706	3541.6854	7592.489	7931.7759	4206.2101	6664.8635	2907.5645	1337.464	4163.3686	9297.4711
F9_Mean	1283.1597	1377.3986	6993.9992	4336.3099	6373.4907	11,028.3681	10,475.5801	5422.9185	11,162.6317	4624.8721	2656.8379	5453.1576	13,558.2981
F9_Std	240.2919	319.962	2141.1304	804.3658	1899.8296	1395.954	1575.4265	677.7583	3234.7729	1123.7032	908.1178	409.1667	3747.3039
F9_Rank	1	2	9	4	8	11	10	6	12	5	3	7	13
F10_Best	3100.4805	3438.9936	4112.4545	7033.0614	3968.2058	8376.048	8112.1966	4191.0162	5982.3824	3988.4912	3581.4997	3467.157	7055.7463
F10_Mean	4658.4277	4924.9309	5041.2709	8163.1076	4918.5357	9299.2026	8639.7047	5219.5294	7667.172	5050.8525	5251.5207	5006.1065	8567.5447
F10_Std	641.2676	781.3113	528.373	458.9554	728.4342	441.962	317.8319	540.6381	778.5117	649.244	1571.3204	736.7535	615.6966
F10_Rank	1	3	5	10	2	13	12	7	9	6	8	4	11
F11_Best	1164.1733	1195.6455	1175.0398	1528.4463	1152.2845	4950.8622	2139.2411	1408.6792	3963.7798	1186.0415	1360.8838	1345.3002	8423.4528
F11_Mean	1252.4661	1317.6392	1321.0814	1945.987	1236.8845	10,910.4909	2918.4837	1573.2479	11,148.2517	1316.1861	2817.3995	2758.3606	16,224.0939
F11_Std	53.0881	80.7739	85.1461	322.8201	40.3686	2213.6768	551.7394	164.5122	4711.8855	72.8846	1421.1518	1023.8185	4672.0359
F11_Rank	2	4	5	7	1	11	10	6	12	3	9	8	13
F12_Best	380,720.0306	317,543.5093	1,031,407.13	77,588,007.6	469,765.2843	5,173,788,403	199,872,673.3	7731,936.12	71,350,290.9	719,782.7217	4,166,565.504	34,905,457.7	1,627,668,409
F12_Mean	1561,728.697	1,841,033.148	6,384,823.608	651,162,027.2	2,970,184.518	14,488,198,785	453,413,228.4	85,710,721	467,027,402.8	9,467,819.867	131,506,251.1	212,503,010.2	8,395,238,895
F12_Std	906,203.5026	1,372,357.756	5,043,674.507	344,600,665.9	4,839,822.692	3,762,670,961	171,306,972.2	72,294,423.2	399,672,624.3	10,283,139.08	110,441,149.2	402,039,201.9	3,205,793,399
F12_Rank	1	2	4	11	3	13	9	6	10	5	7	8	12
F13_Best	9721.8269	19,541.2271	13,711.0633	732,453.39	6463.461	2,587,045,133	64,708.3922	30,686.0841	641,105.352	6634.5051	63,136.3132	35,926,769.81	263,857,044
F13_Mean	26,205.5984	76,243.3456	93,353.907	13,107,818.81	184,795.6642	11,426,344,973	21,485,413.94	271,767.398	10,923,277.48	45,758.8272	24,826,360.92	87,313,208.79	1,845,877,846
F13_Std	12,173.2641	46,986.8035	57,710.2954	20,744,525.4	805,089.9748	6,299,417,101	12,838,619.86	442,948.972	13,931,645.98	33,343.7964	61,121,492.72	36,772,458.51	2,293,586,101
F13_Rank	1	3	4	8	5	13	9	6	7	2	10	11	12
F14_Best	3395.1004	6597.4919	13,281.432	3078.2751	3444.5925	89,579.7151	17,758.8988	5358.5915	24,343.3834	1875.7337	11,460.5692	349,540.4247	543,037.6831
F14_Mean	16,408.2876	50,155.6157	21,8002.6292	60,387.4536	37,821.2518	16,812,178.8	319,070.1003	284,892.2066	2,402,403.034	20,274.3057	544,230.9834	1,487,362.599	8,084,411.29
F14_Std	9725.7892	37,210.1047	32,8511.3707	65,294.4836	33,011.1839	21,955,872.4	168,974.0116	459,674.37	2,465,248.285	19,203.333	558,820.371	863,153.9565	7,905,442.11
F14_Rank	1	4	6	5	3	13	8	7	11	2	9	10	12
F15_Best	2434.283	5263.943	5286.6385	9136.4446	2170.8622	37,361,990.4	383,312.9962	4751.0459	87,761.4939	2981.0319	44,722.4022	3,210,666.9	2,721,958.7
F15_Mean	7222.0835	38,322.072	36,509.4007	58,968.2418	7452.3986	1,018,825,576	2,882,469.4691	18,792.8926	8,808,427.1	12,975.9179	914,084.733	19,653,176.07	297,921,782.2
F15_Std	4532.4615	21,145.1393	18,518.2873	36,426.832	5312.3188	727,272,945.3	1,549,754.918	6777.6747	17,060,712.7	6912.4801	1,862,031.383	47,791,509.51	321,902,006.3
F15_Rank	1	6	5	7	2	13	9	4	10	3	8	11	12
F16_Best	1849.6716	1905.8525	2561.1434	3033.5354	2059.5926	4960.7998	3352.3268	2762.4346	3268.2084	2514.8698	2226.408	2184.7427	3990.0443
F16_Mean	2520.8821	2550.6039	3137.172	3410.7221	2715.0885	6688.2869	3721.7927	3341.2209	4375.5246	3038.9424	2710.0516	2993.4017	5236.7991
F16_Std	321.1201	368.7625	278.2036	254.8473	326.5646	1248.0354	168.5063	386.2889	606.6879	303.5533	337.9585	397.4229	1392.0316
F16_Rank	1	2	7	9	4	13	10	8	11	6	3	5	12
F17_Best	1806.1727	1865.2271	1987.4651	2140.8962	2055.7958	3083.7704	2170.9069	2023.3961	2238.1118	1951.0046	1834.0888	2011.9593	2364.0509
F17_Mean	2100.4224	2222.789	2436.5361	2645.2379	2384.0493	7863.87	2558.1808	2481.2006	2770.053	2453.9147	2113.7112	2628.5312	3515.8741
F17_Std	173.8327	268.852	290.0032	196.1183	250.9484	9262.1162	141.4288	248.5205	289.3042	268.2397	172.2943	305.5762	1095.6607
F17_Rank	1	3	5	10	4	13	8	7	11	6	2	9	12
F18_Best	19,644.3892	31,545.0869	333,460.6781	56,975.2564	107,547.315	5,200,362.03	3,395,040.464	78,317.2713	1,326,182.63	55,350.726	121,681.566	874,511.598	2,405,370.2
F18_Mean	165,178.6273	389,273.4771	2,221,348.364	283,945.7267	734,238.5551	148,427,264.1	15,199,721.15	412,629.7617	16,258,181.37	398,985.794	3,589,206.946	3,167,632.639	88,463,917.7
F18_Std	116,238.0796	348,971.7168	2,987,551.372	213,264.3107	742,859.0111	164,962,112.3	6,751,348.782	513,197.6018	15,865,060.14	571,122.908	5,950,455.72	2,074,320.602	82,787,319.5
F18_Rank	1	3	7	2	6	13	10	5	11	4	9	8	12
F19_Best	2302.9735	2626.3629	2295.563	19,156.4027	2088.4559	76,316,113.5	56,513.3523	27,449.5468	528,625.664	2167.7407	15,671.0971	3,501,444.111	14,392,032.9
F19_Mean	6832.7444	18,157.4446	50,135.8276	2,498,041.85	10,731.0356	863,112,780.3	5,924,214.058	714,903.8554	15,898,560.15	10,878.0561	705,537.6773	12,320,177.25	674,687,208.7
F19_Std	5387.1762	18,525.0824	63,797.1185	4,179,483.44	7868.049	625,233,361.9	3,785,204.637	759,020.3039	153,45,043.74	8631.6748	921,714.6969	17,594,819.58	625,520,209.7
F19_Rank	1	4	5	8	2	13	9	7	11	3	6	10	12
F20_Best	2092.3018	2112.8715	2293.3019	2378.867	2243.9849	2991.1345	2549.8683	2253.6713	2496.261	2410.3075	2157.2159	2286.5827	2640.7625
F20_Mean	2349.4991	2498.8718	2716.9105	2564.5256	2592.6196	3223.9819	2816.482	2559.8019	2981.4016	2731.9635	2488.4509	2582.9051	3134.9736
F20_Std	169.2614	217.1946	241.8618	115.3147	263.6245	148.1703	122.9502	128.6556	234.4609	178.9871	188.9023	164.8756	253.0821
F20_Rank	1	3	8	5	7	13	10	4	11	9	2	6	12
F21_Best	2340.7735	2322.6954	2370.4112	2557.1976	2421.6024	2647.6143	2551.5491	2451.5458	2537.6287	2444.8137	2348.1428	2399.3349	2634.355
F21_Mean	2371.0543	2392.6075	2476.0894	2603.0036	2501.8969	2770.5567	2578.8173	2542.5027	2648.6078	2488.4967	2413.0431	2463.2304	2723.9644
F21_Std	22.5379	24.2156	41.396	19.3173	33.5798	54.5245	14.1465	47.406	58.7126	37.952	31.778	38.0936	39.5068
F21_Rank	1	2	5	10	7	13	9	8	11	6	3	4	12
F22_Best	2300.0014	2300.0366	2300.0112	8076.1258	2313.8309	7863.1368	9219.2141	2323.1812	3199.7732	2311.908	2389.6387	2313.7099	6893.8728
F22_Mean	2653.2082	5567.0697	6085.2019	9484.3027	4507.2187	9962.8861	9964.0637	4310.3664	8387.1595	5443.904	5306.1437	3876.9075	9479.0548
F22_Std	1087.2921	1721.1214	1628.628	444.5196	2203.614	738.959	364.0907	1998.6797	1878.7489	2281.0084	1743.5972	1951.2381	974.8168
F22_Rank	1	7	8	11	4	12	13	3	9	6	5	2	10
F23_Best	2704.0009	2681.7448	2811.6861	2894.7316	2940.342	3499.7936	2880.4977	2861.2597	2933.9339	2916.6556	2738.835	2798.9884	3204.2286
F23_Mean	2740.5205	2742.6945	2900.8565	2933.4512	3200.3548	3833.065	2912.8393	3011.0408	3137.7113	3057.9124	2804.487	2917.4163	3388.6332
F23_Std	27.0832	24.0445	54.1553	13.6454	161.9055	216.7313	12.6722	90.1387	105.1889	93.9146	58.7364	74.2471	116.1025
F23_Rank	1	2	4	7	11	13	5	8	10	9	3	6	12
F24_Best	2857.4146	2875.0429	2915.2972	3042.9888	3066.5	3595.7282	3027.0674	3011.5341	3095.6208	3037.2368	2886.0646	2950.3778	3335.9357
F24_Mean	2896.6124	2911.0879	3056.177	3071.3282	3207.1834	4301.4962	3068.6737	3244.6102	3298.5817	3198.4094	2965.6764	3089.0476	3545.659
F24_Std	22.6983	25.8794	79.16	13.6372	98.7509	251.1912	12.655	106.6723	100.6395	107.6766	61.8868	65.3996	131.4912
F24_Rank	1	2	4	6	9	13	5	10	11	8	3	7	12
F25_Best	2883.6774	2883.6566	2889.5967	3453.3065	2887.4722	4397.8667	3187.535	2909.8596	3059.498	2901.8959	2949.8285	2931.8755	3671.8397
F25_Mean	2910.043	2895.7667	2948.7053	4128.9299	2916.7185	5398.4304	3491.2412	2978.8286	3236.8421	2966.8413	3022.0894	3087.2562	4602.6525
F25_Std	20.6837	13.615	33.7986	288.3817	29.4147	508.5102	146.2479	29.8201	89.1726	30.6491	71.9307	255.7146	424.5
F25_Rank	2	1	4	11	3	13	10	6	9	5	7	8	12
F26_Best	2800.004	4180.5481	3483.6517	6445.5458	2834.2196	9801.021	6153.5248	3703.2469	6641.7505	3180.7911	3439.6321	4583.2437	8582.5369
F26_Mean	4742.0828	4662.6756	6489.2424	6712.6738	5664.7811	11,910.0881	6401.4144	7141.1346	8894.9029	7754.9867	4961.2624	7202.4927	10,281.2444
F26_Std	1194.0367	256.8689	1006.4741	160.1379	2124.1481	947.5293	170.7881	1435.7389	1310.9862	1310.5937	537.2191	1156.2552	1014.2766
F26_Rank	2	1	6	7	4	13	5	8	11	10	3	9	12
F27_Best	3218.8776	3205.436	3258.0386	3292.8519	3233.5834	3954.0359	3233.7974	3282.3146	3322.0881	3321.6779	3223.6669	3249.0434	3543.0786
F27_Mean	3256.88	3232.6215	3329.859	3330.8613	3487.295	5121.2185	3255.9645	3499.9423	3477.4644	3512.3783	3265.0432	3315.0284	4016.0831
F27_Std	19.6475	15.251	52.7178	21.906	225.1439	579.1803	17.1664	109.1238	134.7464	154.888	27.8887	66.0406	385.2681
F27_Rank	3	1	6	7	9	13	2	10	8	11	4	5	12
F28_Best	3200.9585	3215.3417	3256.2831	3839.3131	3209.378	6612.7321	3542.4271	3311.4169	3508.1896	3247.7808	3344.5167	3340.0375	5442.3905
F28_Mean	3234.4864	3257.5043	3320.0865	5308.1523	3244.3537	7875.0428	3814.9043	3415.2796	3910.6755	3312.2048	3461.7024	3492.7689	6557.508
F28_Std	24.0417	32.4207	45.1604	1159.947	27.0636	670.5757	457.733	100.3039	249.0302	32.9647	65.2346	238.4214	780.3821
F28_Rank	1	3	5	11	2	13	9	6	10	4	7	8	12
F29_Best	3504.9622	3472.6914	3862.1975	3888.6695	3656.0831	4989.5138	4498.7683	3904.0941	4657.8584	3944.9812	3607.6881	3479.0091	5079.4719
F29_Mean	3886.0489	4003.293	4311.3918	4427.4571	4183.1916	9630.9879	4907.3246	4843.7188	5543.1906	4819.9595	3987.5993	4462.349	6860.0672
F29_Std	192.2008	284.4441	246.0436	337.1563	271.3288	3912.8556	211.3126	521.3141	445.8885	498.5145	215.6117	342.5997	1088.1623
F29_Rank	1	3	5	6	4	13	10	9	11	8	2	7	12
F30_Best	14,816.9227	25,268.8826	57,386.7756	923,170.1682	25,088.3656	297,852,197	903,758.8879	1,350,896.32	13,108,183.9	54,028.4844	2,209,067.939	6,414,024.65	73,232,495.5
F30_Mean	38,901.3816	82,031.2778	494,883.8104	9,002,097.077	154,606.25	2,248,497,640	3,514,261.213	10,712,842.1	68,351,324.4	530,699.626	10,014,647.3	17,486,485.29	575,206,000.8
F30_Std	18,329.5931	467,48.8136	369,202.1287	8,096,813.629	119,357.06	1,284,773,360	1,554,894.138	11,369,924.07	63,113,680.8	601,079.462	8,464,769.39	7,032,005.78	550,847,465.5
F30_Rank	1	2	4	7	3	13	6	9	11	5	8	10	12
Paired rank +/=/−		25/0/4	29/0/0	29/0/0	28/0/1	29/0/0	28/0/1	29/0/0	29/0/0	29/0/0	29/0/0	29/0/0	29/0/0
Avg. rank	1.21	2.83	5.76	8.34	4.55	12.52	8.62	6.76	10.31	5.62	5.28	7.17	12.03
Overall rank	1	2	6	9	3	13	10	7	11	5	4	8	12

**Table 4:** Wilcoxon signed-rank test results for the EESPPO compared to the other 12 algorithms.

Algorithms	Wilcoxon Test *p*-Value	Significant
EESPPO-PPO	4.0715 × 10^−5^	Yes
EESPPO-SGA	1.1267 × 10^−6^	Yes
EESPPO-HSO	2.0534 × 10^−7^	Yes
EESPPO-PSO	5.6033 × 10^−6^	Yes
EESPPO-FLO	3.9766 × 10^−9^	Yes
EESPPO-DE	9.5497 × 10^−8^	Yes
EESPPO-HO	7.2367 × 10^−7^	Yes
EESPPO-WOA	3.6453 × 10^−8^	Yes
EESPPO-KEO	1.7344× 10^−6^	Yes
EESPPO-GWO	2.9209 × 10^−6^	Yes
EESPPO-IVYPSO	5.4579 × 10^−7^	Yes
EESPPO-FDB-AGSK	6.7488 × 10^−9^	Yes

**Table 5:** The optimal value, average computational time, and the specific parameter settings corresponding to the optimal solution for each algorithm.

Algorithm	Optimal Value	d	D	N	ACTs
EESPPO	0.0127	0.0517	0.3574	11.2472	0.1726
PPO	0.0127	0.0519	0.3626	10.96	0.1662
SRA	0.0127	0.0518	0.3589	11.163	0.2968
HSO	0.0127	0.05	0.3166	14.2301	0.1571
PSO	0.0127	0.0521	0.3657	10.7808	0.1477
FLO	0.0127	0.0531	0.3907	9.5663	0.3004
DE	0.0127	0.0518	0.3597	11.126	0.1682
HO	0.0127	0.0516	0.3547	11.4175	0.66
WOA	0.0127	0.0516	0.3549	11.3987	0.151
KEO	0.0127	0.0515	0.3515	11.6026	0.1904
GWO	0.0127	0.0514	0.3494	11.741	0.1816
IVYPSO	0.0127	0.0523	0.3717	10.4602	0.2272
FDB_AGSK	0.0127	0.0514	0.3504	11.6685	0.2181

**Table 6:** The mean fitness, median fitness, best value, worst value, and standard deviation of each algorithm.

Algorithm	Mean	Median	Best	Worst	Std
EESPPO	0.0127	0.0127	0.0127	0.0127	0
PPO	0.0129	0.0127	0.0127	0.0154	0.0007
SRA	0.0134	0.013	0.0127	0.0168	0.001
HSO	0.0137	0.0132	0.0128	0.0176	0.0011
PSO	0.0138	0.0127	0.0127	0.0305	0.0033
FLO	9714.88	2726.88	0.0127	55,815.07	13,657.58
DE	0.0128	0.0127	0.0127	0.0138	0.0002
HO	0.0129	0.0128	0.0127	0.0155	0.0005
WOA	0.0138	0.0131	0.0127	0.0178	0.0016
KEO	0.0129	0.0127	0.0127	0.016	0.0006
GWO	0.0128	0.0128	0.0127	0.0135	0.0002
IVYPSO	51.2745	0.0144	0.0127	580.6243	147.7679
FDB_AGSK	0.0134	0.0132	0.0127	0.0154	0.0008

**Table 7:** The optimal value, average computational time, and the specific parameter settings corresponding to the optimal solution for each algorithm.

Algorithm	Optimal Value	L	r	D	ACTs
EESPPO	1,677,759.2755	24.469	1.1587	20	0.2947
PPO	1,677,759.2755	24.469	1.1587	20	0.3437
SGA	1,677,759.2755	24.469	1.1587	20	0.8568
HSO	1,677,759.3459	24.47	1.1592	20	0.3107
PSO	1,677,759.2755	24.469	1.1587	20	0.3805
FLO	1,677,759.2755	24.469	1.1587	20	0.6083
DE	1,677,759.2755	24.469	1.1587	20	0.3561
HO	1,677,759.2755	24.469	1.1587	20	0.5696
WOA	1,677,759.2755	24.469	1.1587	20	0.3119
KEO	1,677,759.2755	24.469	1.1587	20	0.3471
GWO	1,677,759.2763	24.4704	1.1587	20	0.3241
IVYPSO	1,677,759.2755	24.469	1.1587	20	0.5541
FDB_AGSK	1,677,759.2755	24.469	1.1587	20	0.3024

**Table 8:** The mean fitness, median fitness, best value, worst value, and standard deviation of each algorithm.

Algorithm	Mean	Median	Best	Worst	Std
EESPPO	1,677,759.2755	1,677,759.2755	1,677,759.2755	1,677,759.2755	0
PPO	1,677,759.2755	1,677,759.2755	1,677,759.2755	1,677,759.2755	0
SGA	1,677,759.2756	1,677,759.2755	1,677,759.2755	1,677,759.2774	0.0003
HSO	1,677,760.8028	1,677,760.4378	1,677,759.3459	1,677,764.9512	1.3693
PSO	1,686,260.0405	1,677,759.2755	1,677,759.2755	1,762,766.9254	25,938.2632
FLO	1,680,592.8639	1,677,759.2755	1,677,759.2755	1,762,766.9254	15,520.2025
DE	1,711,031.4685	1,677,759.2755	1,677,759.2755	2,675,925.0653	182,239.3064
HO	167,778.0719	1,677,759.2755	1,677,759.2755	1,678,323.1659	102.9518
WOA	1,677,759.2759	1,677,759.2757	1,677,759.2755	1,677,759.2777	0.0006
KEO	1,677,759.2755	1,677,759.2755	1,677,759.2755	1,677,759.2755	0
GWO	1,677,759.3378	1,677,759.3219	1,677,759.2763	1,677,759.4862	0.0556
IVYPSO	1,677,790.4382	1,677,759.2755	1,677,759.2755	1,678,257.1008	118.8391
FDB_AGSK	1,750,150.823	1,685,732.6774	1,677,759.2755	2,675,925.0653	251,673.876

**Table 9:** The optimal value, average computational time, and the specific parameter settings corresponding to the optimal solution for each algorithm.

Algorithm	Optimal Value	h	l	t	b	ACTs
EESPPO	1.6702	0.1988	3.3373	9.1918	0.1988	0.2069
PPO	1.6705	0.1987	3.3391	9.1923	0.1988	0.1978
SRA	1.6776	0.1921	3.4702	9.1916	0.1989	0.3357
HSO	1.8209	0.2149	3.2869	8.6758	0.2291	0.1868
PSO	1.6703	0.1988	3.3371	9.1923	0.1988	0.1779
FLO	2.5521	0.1767	5.3054	6.5879	0.3872	0.3613
DE	1.6702	0.1988	3.3372	9.192	0.1988	0.1988
HO	2.1621	0.1673	6.1217	8.2922	0.2458	0.3751
WOA	1.7945	0.1959	3.4383	9.0321	0.2176	0.1819
KEO	1.6702	0.1988	3.3372	9.192	0.1988	0.1941
GWO	1.6716	0.1988	3.3433	9.1906	0.199	0.1855
IVYPSO	1.704	0.1884	3.4833	9.4139	0.1979	0.2308
FDB_AGSK	2.6324	0.1595	5.3363	6.3424	0.4208	0.1801

**Table 10:** The mean fitness, median fitness, best value, worst value, and standard deviation of each algorithm.

Algorithm	Mean	Median	Best	Worst	Std
EESPPO	1.6702	1.6702	1.6702	1.6702	0
PPO	1.676	1.6742	1.6705	1.6952	0.0057
SRA	1.9687	1.8294	1.6776	3.0001	0.3046
HSO	1.9129	1.8973	1.8209	2.1619	0.0788
PSO	1.754	1.7508	1.6703	2.2829	0.1217
FLO	3.6488	3.6259	2.5521	5.4148	0.6348
DE	1.6703	1.6703	1.6702	1.6705	0.0001
HO	2.8154	2.7141	2.1621	3.5651	0.4279
WOA	2.7468	2.5743	1.7945	5.7514	0.885
KEO	1.8611	1.6948	1.6702	2.741	0.3383
GWO	1.6744	1.6734	1.6716	1.6943	0.0042
IVYPSO	2.5132	2.4244	1.704	3.6757	0.558
FDB_AGSK	4.0473	3.6959	2.6324	7.3029	1.1841

**Table 11:** The optimal value, average computational time, and the specific parameter settings corresponding to the optimal solution for each algorithm.

Algorithm	Optimal Value	e_1_	e_2_	e_3_	e_4_	e_5_	e_6_	e_7_	ACTs
EESPPO	2994.2343	3.499	0.7	17	7.3	7.7152	3.3505	5.2867	0.2055
PPO	2994.2343	3.499	0.7	17	7.3	7.7152	3.3505	5.2867	0.1967
SRA	2994.242	3.499	0.7	17	7.3005	7.7153	3.3505	5.2867	0.3308
HSO	3006.2391	3.5004	0.7	17	7.3005	8.1348	3.3564	5.2882	0.1829
PSO	3003.5697	3.499	0.7	17	8.3	7.7152	3.3525	5.2867	0.1742
FLO	3237.2145	3.4847	0.7	17.9735	8.0617	7.9704	3.3931	5.2961	0.3531
DE	2994.2343	3.499	0.7	17	7.3	7.7152	3.3505	5.2867	0.1933
HO	2883.2928	3.5211	0.6847	16.6272	7.8845	7.8433	3.36	5.2876	0.7333
WOA	3002.7054	3.4982	0.7	17	7.3	8.0162	3.3505	5.2893	0.5094
KEO	2994.2343	3.499	0.7	17	7.3	7.7152	3.3505	5.2867	0.1866
GWO	3000.5888	3.5033	0.7002	17.0053	7.3261	7.7324	3.358	5.2876	0.1839
IVYPSO	2997.6166	3.4991	0.7	17	7.3937	7.7389	3.3564	5.2875	0.2255
FDB_AGSK	3012.3585	3.4988	0.7	17	7.3	7.7608	3.3505	5.3135	0.1728

**Table 12:** The mean fitness, median fitness, best value, worst value, and standard deviation of each algorithm.

Algorithm	Mean	Median	Best	Worst	Std
EESPPO	2994.2343	2994.2343	2994.2343	2994.2343	0
PPO	2994.2771	2994.2354	2994.2343	2995.0728	0.1617
SRA	2996.8122	2996.2951	2994.242	3001.3323	2.321
HSO	3018.7289	3019.3416	3006.2391	3028.1878	5.0174
PSO	3209.7622	3158.4842	3003.5697	5605.6447	461.1295
FLO	10,170.9445	9663.4359	3237.2145	21,098.7741	5070.7181
DE	2994.2343	2994.2343	2994.2343	2994.2343	0
HO	3000.0917	3009.3922	2883.2928	3106.7407	50.3322
WOA	3429.9515	3114.3264	3002.7054	5276.6528	713.7096
KEO	2994.2343	2994.2343	2994.2343	2994.2343	0
GWO	3009.7286	3009.9861	3000.5888	3016.6293	4.3098
IVYPSO	4673.6695	3078.6276	2997.6166	21,879.1688	4632.4866
FDB_AGSK	4910.3991	4289.5975	3012.3585	16,117.5827	2506.446

## Data Availability

All data used and/or analyzed during this research are openly available and can be accessed freely. If needed, they can be requested from the corresponding author.
